# The Fatty Acid-Based Erythrocyte Membrane Lipidome in Dogs with Chronic Enteropathy

**DOI:** 10.3390/ani11092604

**Published:** 2021-09-05

**Authors:** Paolo Emidio Crisi, Alessia Luciani, Morena Di Tommaso, Paraskevi Prasinou, Francesca De Santis, Chryssostomos Chatgilialoglu, Marco Pietra, Fabio Procoli, Anna Sansone, Maria Veronica Giordano, Alessandro Gramenzi, Carla Ferreri, Andrea Boari

**Affiliations:** 1 Veterinary Teaching Hospital, Faculty of Veterinary Medicine, University of Teramo, 64100 Teramo, Italy; pecrisi@unite.it (P.E.C.); aluciani@unite.it (A.L.); mditommaso@unite.it (M.D.T.); pprasinou@unite.it (P.P.); fdesantis@unite.it (F.D.S.); mvgiordano@unite.it (M.V.G.); aboari@unite.it (A.B.); 2 ISOF, Consiglio Nazionale delle Ricerche, 40129 Bologna, Italy; chryssostomos.chatgilialoglu@isof.cnr.it (C.C.); anna.sansone@isof.cnr.it (A.S.); 3 Department of Veterinary Medical Sciences, Alma Mater Studiorum—University of Bologna, Ozzano dell’Emilia, 40064 Bologna, Italy; marco.pietra@unibo.it; 4 Ospedale Veterinario I Portoni Rossi, Zola Predosa, 40069 Bologna, Italy; fabio.procoli@portonirossi.it

**Keywords:** canine chronic enteropathy, non-responsive enteropathy, protein-losing enteropathy, membrane fatty acids, lipidomic profile, red blood cell membrane lipidome

## Abstract

**Simple Summary:**

Molecular-based approaches are rapidly developing in medicine for the evaluation of physiological and pathological conditions and for the discovery of new biomarkers in prevention and therapy. Membrane fatty acid-based lipidomic analysis in healthy animals provides a benchmark to study disease conditions and was useful to evidence significant differences in dogs affected by chronic enteropathy. Such molecular information might have the potential to become a useful tool in the assessment of canine chronic enteropathy, being connected with nutritional and metabolic status of the subjects, as well as it may reflect “gut health” and suggest appropriate intervention by “lipid therapy”.

**Abstract:**

Canine chronic enteropathies (CEs) are inflammatory processes resulting from complex interplay between the mucosal immune system, intestinal microbiome, and dietary components in susceptible dogs. Fatty acids (FAs) play important roles in the regulation of physiologic and metabolic pathways and their role in inflammation seems to be dual, as they exhibit pro–inflammatory and anti–inflammatory functions. Analysis of red blood cell (RBC) membrane fatty acid profile represents a tool for assessing the quantity and quality of structural and functional molecular components. This study was aimed at comparing the FA membrane profile, determined by Gas Chromatography and relevant lipid parameter of 48 CE dogs compared with 68 healthy dogs. In CE patients, the levels of stearic (*p* < 0.0001), dihomo–gamma–linolenic, eicosapentaenoic (*p* = 0.02), and docosahexaenoic (*p* = 0.02) acids were significantly higher, and those of palmitic (*p* < 0.0001) and linoleic (*p* = 0.0006) acids were significantly lower. Non-responder dogs presented higher percentages of vaccenic acid (*p* = 0.007), compared to those of dogs that responded to diagnostic trials. These results suggest that lipidomic status may reflect the “gut health”, and the non–invasive analysis of RBC membrane might have the potential to become a candidate biomarker in the evaluation of dogs affected by CE.

## 1. Introduction

Canine chronic enteropathies (CEs) are inflammatory processes that affect the gastrointestinal tract of dogs and, according to the response to a subsequent therapeutic trial, are represented by food-responsive enteropathy (FRE), antibiotic-responsive enteropathy (ARE), and immunosuppressant-responsive enteropathy (IRE). Dogs not responding to treatment are categorized as having non-responsive enteropathy (NRE) [1,2].

In addition to this classification, dogs with loss of protein across the gut are sub-grouped as suffering from protein-losing enteropathy (PLE), highlighting the more guarded prognosis and variable response to therapy for this particular form of CE, compared to dogs with normal serum albumin concentration [3,4,5].

Chronic enteropathies are believed to represent a multifactorial immune-mediated disease, resulting from complex interplay between the mucosal immune system, intestinal microbiome, and dietary components in genetically susceptible dogs, but exact pathogenesis remains largely unknown [2,6,7,8].

In both humans and dogs, the chronic inflammatory state of the gut is associated with increased eicosanoids, including prostaglandins and leukotrienes, which are derived from the metabolism of the ω-6 polyunsaturated fatty acid (PUFA) arachidonic acid (AA) after its detachment from cell membranes by the activity of phospholipase A_2_ [9]. Fatty acids and the resulting metabolites such as eicosanoids are implicated in multiple signaling cascades involved in inflammation, including vascular permeability, edema, chemotaxis, tissue damage, and release of pro-inflammatory cytokines [10,11]; moreover, fatty acids incorporated into phospholipids of the membrane bilayer are recognized as key components of multiple signal transduction cascades, including those associated with the activation and resolution of inflammation [12]. As matter of fact, eicosanoids formed from the long chain PUFA residues of membrane phospholipids, the types of fatty acids forming cell membranes, and the balance between PUFA ω-6 and ω-3 are critical to regulating the signaling outcome [9]. The pro- and anti-inflammatory activities can also be evaluated by calculating the ω-6/ω-3 ratio, taking into account that essential and semi-essential PUFAs are in turn connected to nutritional intakes [11,12]. At the same time, membrane organization, structure, and properties, including mucus production and barrier integrity, are finely tuned by the balance between saturated and unsaturated fatty acid types [12]. These considerations led to the development of the fatty acid-based membrane lipidomic approach, which is a valuable tool to individuate unbalances and establish the strategies called “membrane lipid replacement therapy” [13] and nutrilipidomics [14]. These natural approaches are based on the membrane characterization by choosing a representative cell type in the body that reports on the status of all tissues, and then on the application of the appropriate lipid therapy, which induces a favorable remodeling of the membrane composition by the well-known Lands cycle. The membrane remodeling is the principal phenomenon involved in adaptation and membrane homeostasis, and is activated in several inflammatory and degenerative conditions, as shown for humans [15,16,17].

In recent years, due to the potential role of fatty acids in gut inflammation, fatty acid profile detection and their manipulation have gained great interest in studies of human inflammatory bowel disease (IBD) [11,18,19]. Fatty acids can be altered in the serum, plasma, urine, fecal, and colonic mucosa samples of IBD patients. Increasing evidence, in both humans and rodents, suggests that inflammation alters lipid utilization in the intestine. Indeed, ulcerative colitis (UC) and active and inactive Crohn’s disease patients had a significantly higher proportion of saturated fatty acids (SFAs) and long-chain ω-3 and ω-6 polyunsaturated fatty acids (PUFAs) in intestinal tissue, with a concomitant decline in monounsaturated fatty acids (MUFAs) [20,21]. At the molecular level, SFAs induce inflammatory signaling by stimulating Toll-like receptors (TLR) 2 and TLR4, whereas PUFAs and, in particular, ω-3 docosahexaenoic acid (DHA) are able to counteract such a cascade [22].

In the veterinary field, this subject has also been recently reported, with significant variances identified in plasmatic and whole blood phospholipid profiles of dogs with IRE and FRE [23], and in dogs with IBD after a hydrolyzed diet [24]; however, no information is available on the erythrocyte membrane lipidome. To choose the representative cell type, it must be considered that red blood cells are the largest contributors to cell numbers in the body and make up the highest number of cells formed every day [25]. Furthermore, during their life of 120 days in both humans and dogs, they come in contact with all the bodily districts, exchanging phospholipids and becoming reporters of the tissue conditions, informing on metabolic, nutritional, and healthy conditions with the contribution of long-term nutritional habits [14,15,26,27]. Although plasma has been considered to follow a lipidomic pattern similar to that of the tissues [28], it is worth recalling that the fatty acid content of plasma can be greatly affected by short-term dietary intake [29,30].

The benchmark of a healthy dog membrane lipidome was reported following the protocol used also for humans, isolating the blood fraction containing erythrocytes [31], and based on this premise, we were interested in characterizing dogs affected by CE, to investigate the molecular differences from the healthy condition. It can be expected that the quantity and quality of fatty acids in the erythrocyte membrane lipidome may mirror the “gut health” in dogs affected by different forms of CE, allowing us to gain new insights related to the balance among fatty acid types that regulate signaling cascades. Diagnostic performance of the erythrocyte lipidome in distinguishing different subtypes of CE (i.e., FRE, ARE, and IRE/NRE) was also evaluated. Finally, we assessed for any possible association between the red blood cell lipidomic profile in CE dogs according to the Canine Chronic Enteropathy Clinical Activity Index (CCECAI), the body condition score (BCS), as well as C-reactive protein (CRP), albumin, folate, and cobalamin levels.

## 2. Materials and Methods

### 2.1. Animals

Dogs with primary chronic gastrointestinal signs presented to Veterinary Teaching Hospitals of the Universities of Teramo and Bologna and to the Veterinary Hospital “I Portoni Rossi”, Italy, between January 2018 and June 2019 and were prospectively enrolled in the study. The project was approved by the Committee on Animal Research and Ethics of the Universities of Chieti-Pescara, Teramo and Experimental Zooprophylactic Institute of AeM (CEISA), Protocol UNICHD12 n. 1168.

Dogs were eligible for the study if they had a history of clinical signs for at least 3 weeks that were consistent with CE, including vomiting, diarrhea, and/or weight loss. Inclusion criteria were parasitological negativity of the copromicroscopic examination (i.e., direct fecal smear evaluation and zinc sulfate centrifugal flotation techniques) performed on 3 fecal samples and exclusion of common extra-gastrointestinal causes of chronic gastrointestinal signs, such as exocrine pancreatic insufficiency or hypoadrenocorticism (i.e., complete blood count, serum chemistry panel, urinalysis, evaluation of basal cortisol or adrenocorticotropic hormone stimulation test if needed, canine Trypsin-like Immunoreactivity, and abdominal ultrasound). Exclusion criteria were recent (<1 month) anti-inflammatory/immunosuppressive treatment, antibiotic treatment, and administration of probiotics. Additionally, dogs that received dietary ω-3 supplementation in the last 4 months were not included in the present study. Dogs already in dietary management were not excluded from the study. The control group was made of 68 healthy dogs, whose fatty acid-based membrane lipidome profile was recently published [31].

### 2.2. Diagnostic Investigations and Therapeutic Trials

At the time of the first visit, each dog included in the study underwent blood sampling for lipidomic analysis after 12 h overnight fasting and before any interventions. The Canine Chronic Enteropathy Clinical Activity Index (CCECAI) [3] and the body condition score (BCS) were recorded for each enrolled patient, and every dog underwent a 5-day course of fenbendazole 50 mg/Kg per *os* once a day, regardless of the fecal tests results.

Based on the diagnosis, dogs affected by chronic enteropathy (CE) were further divided into groups [1,2], as follows. The FRE group included patients with complete remission of the gastrointestinal symptoms within 3 weeks of dietetic trial with an elimination diet or a hydrolyzed protein diet; the ARE group included patients with partial or no response to dietetic trial, but complete remission of the gastrointestinal symptoms within 3 weeks of an antibiotic trial with tylosin at a dose of 15 mg/Kg twice a day; patients that responded neither to diet nor to antibiotics underwent multiple gastrointestinal endoscopic biopsies and were classified as IRE/NRE. Dogs that went into remission on anti-inflammatory/immunosuppressive drugs such as prednisolone or budesonide were classified as IRE, and those dogs that responded poorly to anti-inflammatory/immunosuppressive therapy were classified as NRE. The remission was considered complete if clinical signs resolved or the CCECAI score reduced by ≥75% and maintained for a minimum of 6 weeks, while dogs in the NRE group showed no clinical response to treatment, as assessed by a change in CCECAI scores of <25% or no clinical improvement [32]. The same therapeutic diet was continued throughout the trial in all the CE dogs.

Moreover, regardless of the diagnosis (i.e., FRE, ARE, or IRE/NRE), those patients with low albumin concentration (<2 mg/dL) due to a severe loss of serum proteins into the intestine were further classified into the PLE subgroup.

### 2.3. Fatty Acid-Based RBC Membrane Lipidome Analysis

Whole blood in EDTA as anticoagulant was used to isolate red blood cells and perform membrane lipidome analysis, as previously described [31]. Briefly, starting from a 1 mL whole blood sample, the separation of erythrocytes from plasma, followed by washings and centrifugation for the membrane isolation, finally produces a pellet that was re-suspended in pure water to extract lipids using 2:1 chloroform:methanol as the organic phase [31]. The organic layer was separated and evaporated under vacuum to dryness. A thin layer chromatography using chloroform/methanol/water in a ratio of 65:25:4 was performed to determine the purity of the phospholipid fraction with respect to the presence of residual triglycerides or cholesteryl esters as plasma contaminants that could affect the fatty acid quality and quantity [33]. The phospholipid extract was transesterified at room temperature for 10 min with 0.5 M KOH/MeOH to obtain the corresponding methyl esters (FAMEs) of the fatty acid residues esterified to the glycerol moieties. This chemical transformation was carried out with known procedures, checking for the absence of oxidative and degradation reactions, which could affect the final fatty acid composition. The final steps were the extraction of the FAME using n-hexane, evaporation under vacuum to dryness, and use of the FAME extract to perform gas chromatography (GC) analysis, as described below (see Figure S1 in Supplementary Information).

FAME extract was dissolved in 20 μL of n-hexane and 1 μL was directly injected into the Agilent 7890B GC system equipped with a flame ionization detector and a DB-23 (50%-Cyanopropyl)-methylpolysiloxane capillary column (60 m, 0.25 mm i.d., 0.25 μm film thickness). The initial temperature was 165 °C, held for 3 min, followed by an increase of 1 °C/min up to 195 °C, held for 40 min, followed by a second increase of 10 °C/min up to 240 °C, held for 10 min. The carrier gas was hydrogen, held at a constant pressure of 16.482 psi. In Supplementary Information, there are details of the calibration procedure and a figure of a representative GC run (Supplementary Figures S2 and S3) with the 10 fatty acids identified by appropriate standard references, which are commercially available as reference materials at known concentrations. The quantification procedure was performed as already described [31] and reported in Supplementary Information. The data of the 10 FAs are represented in μg/mL, and each quantity is reported as percentage relative to the cluster as sum of the quantities (100%) that corresponds to >97% of the peaks found in the GC runs (% rel. quant.). The quantities were used to obtain the corresponding lipid indexes, which were derived from performing mathematical operations of sums and ratios using the FA quantities as described in the text and table footnotes.

### 2.4. Evaluation of the Fatty Acid Cluster, Homeostasis Indexes, and Enzymes Activity Indexes

As previously described [31], a cluster of 10 fatty acids, representative of the main fatty acid moieties present in the cell membrane, was chosen. In particular, the cohort of fatty acids included: palmitic (C16:0) and stearic (C18:0) acids as SFAs; palmitoleic (C16:1), oleic (9c, C18:1), and vaccenic (11c, C18:1) acids as MUFAs; linoleic (LA, C18:2), dihomo-gamma-linolenic (DGLA; C20:3), and arachidonic (AA, C20:4) acids as PUFA ω-6; and eicosapentaenoic (EPA, C20:5) and docosahexaenoic (DHA, C22:6) acids as PUFA ω-3. These values were also reported as total fatty acid contents (total SFAs, total MUFAs, total PUFAs, total ω-6, and total ω-3), and lipid indexes were calculated as follows: ω-6/ω-3 ratio, PUFA balance [ω-3/(ω-3 + ω-6)], SFA/MUFA ratio, unsaturation index (UI = MUFA total × 1 + C18:2 × 2 + C20:3 × 3 + C20:4 × 4 + C20:5 × 5 + C22:6 × 6) and peroxidation index (PI = MUFA total × 0.025 + C18:2 × 1 + C20:3 × 2 + C20:4 × 4 + C20:5 × 6 + C22:6 × 8) (38). The elongase-6 activity (EI = C18:0/C16:0), delta-9 desaturase activity (D9DI = 9c, C18:1/C18:0), delta-6 desaturase activity (D6DI = C20:3/C18:2), and delta-5 desaturase (D5DI = C20:4/C20:3) were calculated as precursors to fatty acid ratios, as described in several papers [34,35]. The gas chromatographic method was able to satisfactorily separate all 10 of the fatty acids, without superimposition of other peaks, in particular, the positional and geometrical isomers of the unsaturated fatty acids, using conditions that were presented in previously reported papers [15,34,36]. The quantitation of the fatty acids was executed by a known calibration procedure, as reported in Supplementary Information.

### 2.5. Statistical Analysis

Computer software was used to perform the analysis (GraphPad Prism version 6.01, GraphPad Software, La Jolla, CA, USA). All data were evaluated using a standard descriptive statistic and reported as the mean ± SD, or as median and range (minimum–maximum), based on their distribution. Normality was checked using the D’Agostino Pearson test. A comparison between 2 groups was performed using the unpaired t-test or the Mann–Whitney test, while a comparison among more than 2 groups was completed using the ANOVA or a Kruskal–Wallis test and post hoc tests (Student–Newman–Keuls test or Dunn’s test). A regression analysis was used to evaluate the correlation between fatty acid percentages, fatty acid indexes, or enzyme activity indexes and CCECAI and BCS, as well as CRP, folate, and cobalamin levels of CE dogs. The threshold of statistical significance was set at *p  <*  0.05.

## 3. Results

### 3.1. Study Population

The characteristics of the 117 included dogs are summarized in Table 1; 48 of these were diagnosed with CE, while 68 were healthy dogs enrolled as control group, the latter characteristics being previously published [31]. Detailed data about dogs belonging to the cohort of CE are presented in Supplementary Table S1.

The CE group comprised 17 females (7 spayed) and 31 males (1 neutered), with a median age of 46 months (range 4–144). Dogs belonging to the control group were 38 females (12 spayed) and 30 males (6 neutered) with a median age of 41 months (2–156). Twenty-eight dogs responded to the dietary trial and were classified as FRE, 5 failed a dietary trial, but responded to tylosin and were diagnosed with ARE. Twelve dogs failed to respond to sequential dietetic and antimicrobial trials, and these patients either responded well to immunosuppressive treatment (*n* = 9) and were classified as IRE or poorly responded (NRE; *n* = 3). Clinical and clinicopathological characteristics of dogs affected by FRE, ARE, and IRE/NRE are summarized in Table 2 and other characteristics are reported in Supplementary Table S1.

The median CCECAI value in CE dogs was 5 (1–19), with a median albumin level of 3.1 (0.6–3.8). Folate and cobalamin levels were available in 37/48 CE dogs, with a median value of folates of 8.7 μg/L (1.0–24.0 μg), and a median cobalamin level of 381.5 ng/L (150–994 ng/L) (150 ng/L represented the lower detection limit for cobalamin methods used, i.e., chemiluminescence). In particular, 12 dogs had folate and cobalamin levels within the reference interval (i.e., 6.5–11.5 μg/L and 250.0–730.0 ng/L, respectively), 4 dogs had both folate and cobalamin below the reference intervals, while 1 dog had both folate and cobalamin above the reference intervals, 4 dogs had normal folate but were hypocobalaminemic, 1 dog with normal folate had high cobalamin concentrations, and 1 dog with low folate levels had high cobalamin concentrations. Fourteen dogs with normal cobalamin levels had altered folates (7 seven dogs had folate below the reference interval and 7 had folate levels above the reference intervals).

Nine out of 48 CE dogs had serum albumin levels <2 mg/dL and were further classified as PLE. Among these, one dog responded to dietary management, one dog responded to tylosin, four dogs to immunosuppressant therapy, while three dogs were classified as NRE.

### 3.2. Fatty Acid-Based RBC Membrane Analysis

**Figure 1:**
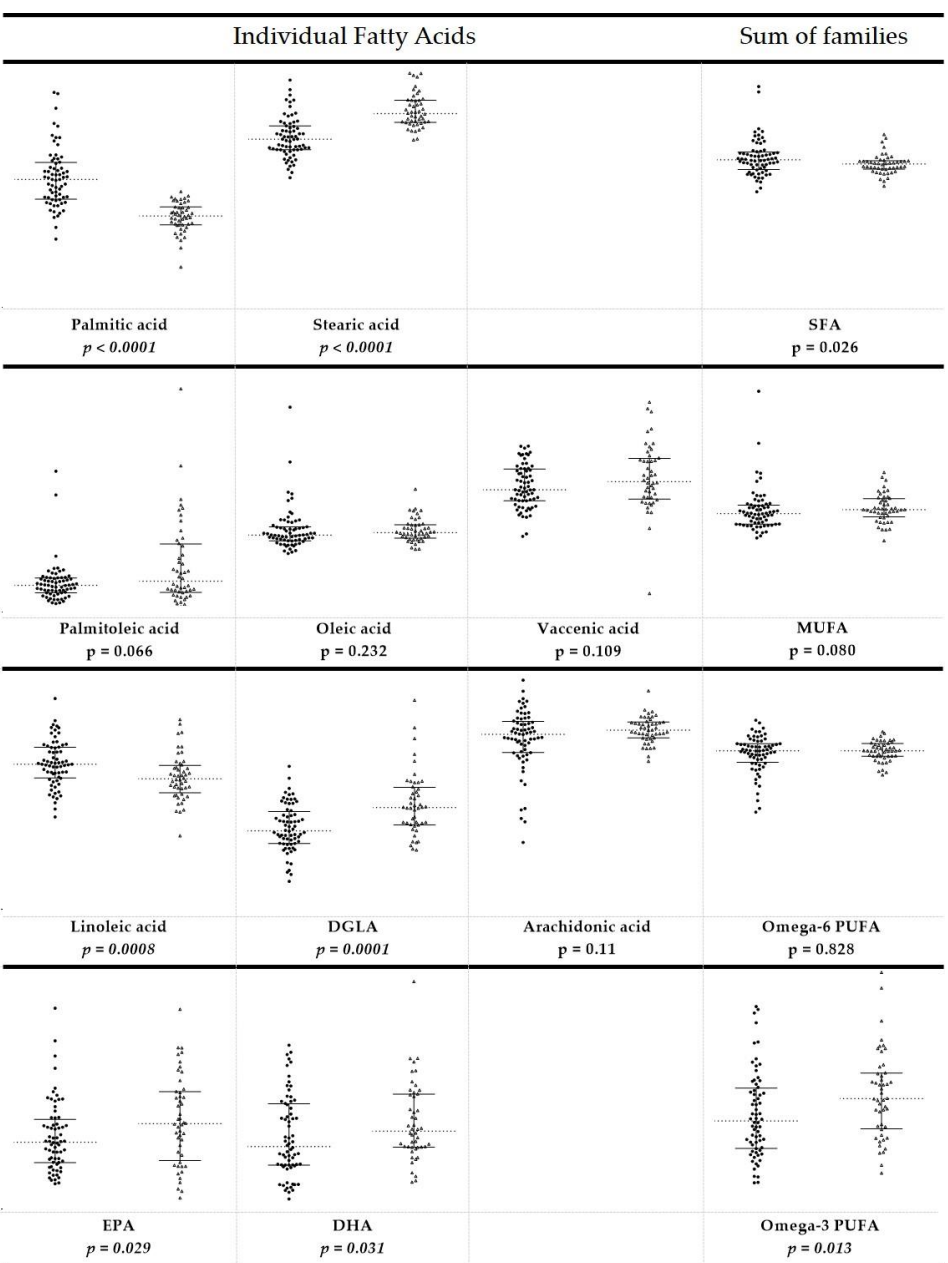
Scatter dot plot of the individual fatty acids and the corresponding families in the population of dogs affected by chronic enteropathy (*n* = 48, triangles, right) compared to the values obtained from healthy dogs (*n* = 68, circles, left). Each member of the fatty acid family is given in a row, the last column being the sum of the corresponding fatty acid family. Horizontal dashed lines represent the median values. The end points of the vertical lines show interquartile range. The *p*-values are reported, with values significantly different in italic.

**Figure 2:**
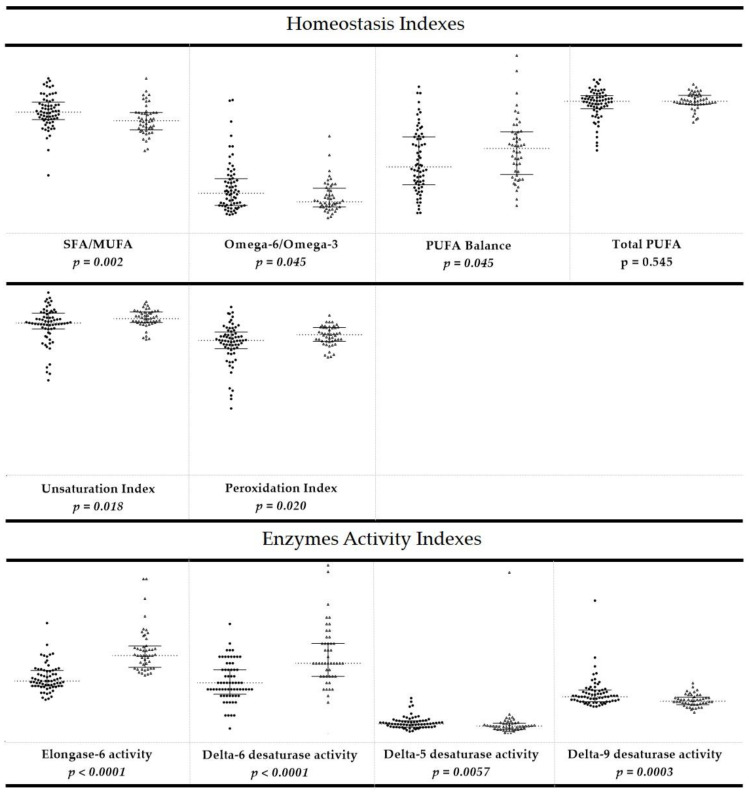
Scatter dot plot of the calculated homeostasis indexes and enzyme activity indexes in the population of dogs affected by chronic enteropathy (*n* = 48, triangles, right) compared to the values obtained from healthy dogs (*n* = 68, circles, left). Horizontal dashed lines represent the median values. The end points of the vertical lines show interquartile range. The *p*-values are reported, with values significantly different in italic.

The values of single fatty acids, total fatty acid contents (total SFAs, total MUFAs, and total PUFAs), ratio between the families (SFA/MUFA, ω-6/ω-3), and indexes (UI, PI, and PUFA balance; D9DI EI, D6DI, and D5DI) of CE and healthy dogs are reported in the Supplementary Table S2. The reference intervals were reported as interquartile (IQ) range (Table 3). Figure 1 and Figure 2 also report the values of the fatty acids, the corresponding FA families, and calculated indexes.

It was gratifying to see that the FA cluster showed significant changes for CE dogs compared to healthy animals: (a) lower values of palmitic acid (*p* < 0.0001) and higher levels of stearic acid (*p* < 0.0001) compared to healthy dogs, with an overall reduction in total SFAs (*p* = 0.026); (b) ω-6 PUFA in CE dogs showed a reduced content of linoleic acid (*p* = 0.0008) and an increase of dihomo-gamma-linolenic acid (*p* = 0.0001) compared to healthy dogs; (c) ω-3 PUFA in CE dogs with higher EPA (*p* = 0.029) and DHA (*p* = 0.031) contents, as well as an increased content of total ω-3 PUFA (*p* = 0.013). On the other hand, no differences were observed between healthy and CE dogs for each MUFA (palmitoleic, oleic, and vaccenic acids) and total MUFA values, as well as for arachidonic acid levels and total content of ω-6 PUFA.

In CE dogs, the differences included membrane homeostasis index patterns compared to healthy dogs (Table S2). In particular, CE dogs had reduced values of ω-6/ω-3 (*p* = 0.045) and SFA/MUFA (*p* = 0.002) ratios, and increased PUFA balance (*p* = 0.045), UI (*p* = 0.018), and PI (*p* = 0.02). Moreover, CE dogs showed significant differences in enzymatic activity indexes; in particular, elongase (*p* < 0.0001) and delta-6 desaturase (*p* < 0.0001) indexes were found to be increased, while delta-5 (*p* = 0.0057) and delta-9 (*p* = 0.0003) desaturase indexes were lower when compared to healthy dogs.

No significant differences were observed in fatty acid contents, membrane homeostasis indexes, and enzyme activity indexes among dogs affected by different forms of CE (i.e., FRE, ARE, IRE/NRE) (Supplementary Table S3), or between CE dogs with and without PLE (Supplementary Table S4).

The red blood cell membranes of dogs that did poorly or did not respond to anti-inflammatory/immunosuppressive therapy (i.e., NRE) presented higher percentages of total MUFAs (*p* = 0.0317), in particular, vaccenic acid (*p* = 0.0077), compared to those of dogs that responded to diagnostic trials (i.e., FRE, ARE, and IRE) (Supplementary Table S5).

As far as the correlations with other biochemical parameters were concerned, they are reported in Table 4. Negative correlations were observed for: (a) the levels of cobalamin and vaccenic acid (*p* = 0.028; r = −0.362) and D6DI (*p* = 0.029; r = −0.358); (b) CCECAI and ω-6 PUFA levels (*p* = 0.040; r = 0.300); and (c) folate and oleic acid (*p* = 0.031; r = −0.355), vaccenic acid (*p* = 0.035; r = −0.347), total MUFAs (*p* = 0.026; r = −0.365), and D6DI (*p* = 0.039; r = −0.340). Positive correlations were found as follows: (a) folate levels with ω-6 PUFA (*p* = 0.045; r = −0.331), total PUFAs (*p* = 0.024; r = 0.369), and D5DI (*p* = 0.048; r = 0.327) and (b) CRP with palmitic acid (*p* = 0.049; r = −0.317) (Supplementary Figure S4). Body Condition Score (BCS) and serum albumin levels did not correlate with fatty acid contents or membrane homeostasis indexes.

## 4. Discussion

In this study, we compared the fatty acid cohort found in the red blood cell membrane lipidome in dogs affected by chronic enteropathy with the reference values obtained for healthy dogs and previously described [31].

We chose to have a cluster of 10 fatty acids, representative of each family (i.e., saturated, monounsaturated, and polyunsaturated), taking into account that: (a) their quantities are relevant to form most of the cell membranes, and without them membranes cannot be formed; (b) they play predominant biochemical roles; and (c) they have very well-known synthetic and metabolic pathways as well as biological effects. On the other hand, the choice of these elements did not exclude the importance of other fatty acids.

In general, we believed that the information from the membrane lipidome could help connect the clinical and the nutritional status of the patients, as happens with humans. The fatty acid cohort, here obtained from the erythrocyte membranes, belonged to the most representative fatty acid types in terms of metabolism (liponeogenesis and delta-9 desaturase activities, such as SFAs and MUFAs) and nutrition, namely, the essential PUFAs, that are then transformed by further elongation and delta-6 and delta-5 desaturase enzymatic activity into eicosanoids, as explained in the Introduction. In the present study, CE dogs showed consistent differences from the healthy dogs, mainly regarding the SFA-MUFA pathway and the PUFA levels, which were examined in detail for their metabolic significance.

Starting with the SFA family, decreased palmitic acid and increased stearic acid levels in the RBC membrane lipidome of CE dogs were already observed in RBCs of humans affected by chronic gastrointestinal disorders, such as Crohn’s disease and celiac disease [37,38]. These results often indicate the hyperactivation of the elongation pathway (see also the significant increase in the corresponding enzymatic activity, Table S2) in animals as in humans. However, the SFA content results were significantly reduced by the significant decrease in palmitic acid (*p* < 0.0001). It is worth noting that the membrane status examined in the RBC lipidome represented the closest indication of the tissue content [39,40,41,42], therefore the SFA diminution assumes a metabolic significance in animals, connected to the previously known SFA diminution in erythrocyte membranes reported for ulcerative colitis [39] and celiac disease [38]. On the other hand, it was observed that increased stearic acid levels did not induce any increase in the monounsaturated levels, as reflected by the delta-9 desaturase index that was significantly lower in CE dogs (cf. Table S2). As matter of fact, in active human ulcerative colitis, the gene expression of such desaturase was reported to be reduced [43] and the liver expression of delta-9 desaturase was dramatically inhibited in colitis mice [44,45].

As the fatty acid cluster also expressed the remodeling of the families within the functional compartment of the membrane, it is worth noting that the significant SFA diminution was accompanied by a complementary significant increase in PUFA, such as DGLA (ω-6), EPA, and DHA (ω-3). Such a result also contains important nutritional information as, as explained in the Introduction, PUFAs are essential and derive from food intakes, and the balance between ω-6 and ω-3 PUFAs is informative about the uptake of adequate sources of these fatty acids. The RBC membranes can give precise evaluation of the PUFA content in other tissues as reported for a murine model fed different diets [40]. Palmitic acid was implicated in the pathogenesis of human IBD [46], and its role in intestinal health has been discussed, this fatty acid having been implicated as a ligand of Toll-like receptors (TLR4) [47], whereas down- or upregulation of TLR expressions are found in different canine CE conditions [48,49]. It is also important at this point to consider that membrane phospholipid composition also influences the balance of lipid mediators involved in intestinal diseases; therefore, mediators from palmitic acid, such as palmitoylethanolamide [50,51] and, from PUFA, for example, docosahexanoyl serotonin [52], can be influenced by the change in the precursor’s levels in the membrane.

We believe that the concept of the membrane fatty acid cluster can contribute to creating a comprehensive vision of its structural and functional roles in health and diseases by considering the levels of SFAs balanced by MUFA and PUFA levels as the result of epigenetic factors, such as nutrition, stress, and environment exposures.

Considering the PUFA levels, in the ω-6 family linoleic acid was found significantly lower and DGLA was significantly higher than the levels of these two fatty acids found in healthy dogs. This result indicated the similarity of dog RBC lipidome to the RBC lipidome in human IBD and in celiac disease, where the metabolism of linoleic acid supported by the delta-6 desaturase seems to be accelerated more than that of healthy individuals [37,38,53]. In accordance with what has been previously reported in the literature, the concentrations of linoleic acid were significantly lower in the serum of patients with chronic intestinal disease [54,55,56]. Accordingly, the profile of CE dogs showed an hyperactivation of this enzymatic pathway with faster metabolization of linoleic acid to DGLA and a subsequent increase in the delta-6 desaturase activity index. Interestingly, these enzymes are mainly located in the liver and in the intestinal mucosa, and a reduction in linoleic acid levels in patients with severe malabsorption or chronic intestinal disease was hypothesized as being related to a specific enhancement of linoleic acid elongation and desaturation by an increase in desaturase activity [55]. This condition seems to be exacerbated if a concurrent malabsorption of vitamin exists; indeed, the present data demonstrate the significant negative correlation of the D6DI increase with decreased plasmatic concentrations of folate and cobalamin (cf. Table 4). Interestingly, in humans, genetic polymorphisms associated with alterations in the metabolism of long chain PUFAs from dietary linoleic acid and α-linolenic acid have been associated with the risk of developing Crohn’s disease [57,58,59].

Although an effect on the PUFAs involved in the bowel inflammation process might be expected from the eicosanoids derived from arachidonic acid [29,60], in our cohorts, the total ω-6 PUFA and arachidonic levels did not differ between CE and healthy dogs. Given that approximatively two-thirds of CE dogs had a mild activity of intestinal disease based on the CCECAI score, this finding might be expected. On the other hand, it is tempting to speculate that the increased EPA, DHA, and total ω-3 PUFA levels found in the CE dogs of the present study, compared to controls, were coupled with the levels of arachidonic acid, which were not different in the two cohorts. Indeed, the levels of the ω-3 PUFA EPA had an impact on the amount of arachidonic acid in cell membranes and their pro-inflammatory derivates under IBD conditions [61]. In particular, the relationship between EPA and arachidonic acid derived from: (a) the respective biochemical pathways, as the precursors of these omega-3 and omega-6 fatty acids share the same enzymes (delta-6 desaturase, elongase, and delta-5 desaturase), therefore they compete for their formation, and if the quantity of the omega-6 precursor is reduced in the diet, the omega-3 EPA will consequently be predominant; and (b) EPA and arachidonic acid utilize the same enzymes to produce prostaglandin series 3 and 2 that have anti- and pro-inflammatory activity, respectively, therefore they have a biochemical relationship and one controls the effects of the other. It is worth noting also that the debate over the PUFA levels in intestinal disease is still open, considering patterns previously observed in active IBD patients showing a significantly higher fraction of the ω-3 PUFA and DHA [62,63]. Furthermore, the inflammatory process implies that an increased utilization of fatty acids and increased concentrations of DHA could be related to a higher expression of phospholipase A_2_. Indeed, DHA is also responsible of increasing the biosynthesis of PUFAs, which in turn play an anti-inflammatory role [64]. Moreover, prior studies found plasma ω-3 PUFA concentrations increased in patients with Crohn’s disease compared to control patients; moreover, in these patients, total ω-3 PUFA and EPA levels directly correlated with pro-inflammatory circulating cytokine levels [65] and are inversely correlated with tissue cytokines [66]. Interestingly, an inverse correlation between CCECAI and ω-6 PUFA was observed in CE dogs, corroborating a fascinating hypothesis according to which PUFA may be upregulated in mild disease and consumed as disease activity increases [66].

Considering the scenario emerging from the RBC membrane molecular profile of dogs, it can be observed that SFAs, stearic and palmitic acids, and the ω-6 PUFAs, arachidonic and linoleic acids constitute ca. 94% of the total RBC fatty acids. Therefore, the significant changes in palmitic, stearic, and linoleic acids levels found in diseased dogs mirror a relevant metabolic derangement of the main membrane components. On the other hand, in the ω-6 pathway the elevated levels of DGLA assume a meaning if one considers that in the dog metabolism the linoleic acid pathway to gamma linolenic and DGLA exerts the main anti-inflammatory control of the animal, leading to potent anti-inflammatory eicosanoids. In fact, DGLA is metabolized by cyclooxygenase (1 and 2) and arachidonate 15-lipoxygenase into prostaglandins and anti-inflammatory eicosanoids, with the ability to antagonize the synthesis of AA-derived pro-inflammatory eicosanoids [67]. Indeed, since the first historical observation of dermatological canine problems, it should be recalled that, in dogs, ω-3 does not play the same physiological role as in human metabolism for the control of inflammation [68].

The overview gathered from humans and animals on the essential fatty acid intakes and, in general, on the importance of fats is still awaiting the appropriate consideration in experimental and clinical protocols, but we believe that the time for integrating such molecular information has arrived. Considering the knowledge gathered from animals and humans evidencing membrane lipid alteration and effects of dietary conditions [69,70] and the knowledge acquired about the ideal membrane lipid composition of tissues [71], the fatty acid-based approach provides molecular information on the lipid pool and the balance reached in the membrane compartment.

It is also worth noting that membrane homeostasis indexes contribute information of alterations in CE dogs, in particular: (a) the ω-3/ω-6 ratio, which is known to be influenced through diet therapy as an important factor for reducing intestinal inflammatory conditions in humans and animals; and (b) the PUFA balance (ω-3/ω-3 + ω-6) proposed by Abbott and colleagues [40] to evaluate the overall PUFA effects in membranes, which also adds more information about the role of diet therapy and nutraceutical interventions. In particular, these indexes can be useful to evaluate the appropriateness of ω-3 supplementation in canine CE, and it should also be recalled that the increase in DGLA can only be efficiently contained by ω-3 α-linolenic acid, which is the natural substrate of the delta-6 desaturase, balancing the enzymatic conversion of the ω-6 linoleic acid.

Furthermore, considering the biophysical effects and chemical reactivity of the unsaturated fatty acids, unsaturation and peroxidation indexes are useful parameters for estimating the homeostasis of membranes in terms of fluidity and oxidative damage, respectively, while maintaining an appropriate environment for membrane function. Fatty acid unsaturation is considered a parameter by which to evaluate longevity in animal species, and the membrane peroxidation index is inversely related to maximum life span in mammals [72]. In our cohort of CE dogs, both indexes were increased (cf. Table S2), indicating a perturbation of the membrane compartment and suggesting the need for strategies for antioxidant protection. Indeed, oxidative damage in dogs with CE has been recently demonstrated [73], and membrane changes can contribute to this reactivity. The usefulness of antioxidant treatment alongside the traditional therapeutic approaches should be investigated in detail in canine CE, and the membrane lipidome analysis can be useful when tuning the supplementation according to the amelioration of this molecular indicator.

Fatty acid-based membrane lipidomes were also evaluated for the different clinical conditions of CE dogs, but only a few values connected to the specific diagnosis were found (Supplementary Tables S3–S5). Despite the clinical differences among CE dogs, including the specific intestinal absorption condition, the erythrocyte membrane lipidome was able to report a systemic picture of the lipid metabolism that directed attention to specific indicators, such as stearic acid, DGLA, EPA, and DHA, to monitor the therapeutical intervention in chronic enteropathy. Indeed, dogs with loss of protein across the gut have a more guarded prognosis compared to dogs with normal serum albumin concentration [3,4]; however, no differences were highlighted in lipidomic profiles of dogs affected by PLE and dogs with CE without loss of protein. On the other hand, despite a small number, we highlighted that dogs failing to respond to therapeutic trials (i.e., NRE) showed higher levels of vaccenic acid and total MUFAs (Supplementary Table S5). Interestingly, vaccenic acid levels were also inversely correlated with cobalamin concentrations, and hypocobalaminemia was considered a negative prognostic factor in dogs with CE [3] (cf. Table 4). *Cis*-vaccenic acid is obtained from palmitoleic acid by elongases 5 and 6, and both enzymes would seem to be involved in the pathogenesis of gut inflammation. In fact, data of the present study suggest a hyperactivation of elongase 6 in canine CE, while recently, in human UC, elongase 5 was found significantly upregulated in memory CD4+ T cells and follicular helper T cells [74]. An imbalanced elongase 5 activity may result in an alteration of MUFA metabolism, and the accumulation of vaccenic acid, observed in NRE dogs, may affect the membrane fluidity and the signaling to influence T cell proliferation and function, as already observed in humans [75].

Finally, the positive correlation between folate and ω-6 PUFA, and folate and total PUFAs, can also be evaluated in view of the known correlation in humans between folate and PUFA [76,77,78]. It is worth mentioning that cobalamin and folate are essential for methylation reactions, which in turn may influence the transport of PUFAs in red blood cells [79].

We believe that this study, for the first time, shows the relevance of the fatty acid-based RBC membrane lipidome analysis in dogs affected by CE, by comparison with the benchmark offered by the membrane lipidome of healthy dogs. We highlighted differences that correlated with metabolic and signaling pathways of this inflammation-based disease. Our approach, based on the representative cohort of 10 fatty acids present in the erythrocyte membrane, shows promising diagnostic value for determining canine condition. We are perfectly aware that the approach is directed at the fatty acid moieties and not the whole membrane lipidome. The procedure followed in this study ensures that positional and geometrical isomers of unsaturated fatty acids, including MUFAs [34,36] and PUFAs [80,81], can be well separated by gas chromatographic conditions; therefore, the unsaturated fatty acids determined in our cohort do not suffer from artifacts due to peak superimposition. On the other hand, the picture given by the fatty acid distribution of the cohort refers to the fatty acid moieties esterified in the glycerophospholipids and plasmalogens that compose the RBC membrane. More sophisticated procedures, including the use of expensive mass spectrometry facilities, have been described to give the whole composition of membranes [71]; however, we believe that the validity and the applicability of the fatty acid-based RBC membrane analysis, which has been demonstrated in humans, to follow-up on nutritional and metabolic effects of EC is promising in animal health.

## 5. Conclusions

Understanding red blood cell composition in terms of the quantity and quality of fatty acids provides important novel information on the pathogenesis of canine chronic enteropathy and, albeit preliminarily, suggests that lipidomic status may be a mirror of the “gut health”, turning attention toward the molecular aspects of canine CE. Understanding the distribution of erythrocyte membrane fatty acids will provide important insights into the lipid metabolism of dogs affected by this condition as well as become a guide for future biomarker selection. Moreover, given its non-invasiveness, the analysis of the erythrocyte membrane lipidome is a promising candidate as a useful tool to investigate and gather preliminary information when planning interventional studies and follow-up of the patients during treatments.

## Figures and Tables

**Table 1:** Number of males and females, median age (in months), and body weight (in kilograms) with minimum and maximum in brackets of the study dogs. Italic values denote statistical significance.

Variable	Healthy Dogs (*n* = 68)	Chronic Enteropathy (*n* = 48)	*p* Value
Males	30	31	*0.04*
Females	38	17	
Age	41 (2–156)	46 (4–144)	0.83
Bodyweight	22.0 (2.6–43.0)	22.0 (4.0–36.3)	0.43

**Table 2:** Clinical and clinicopathological characteristics of dogs affected by chronic enteropathy. Italic values denote statistical significance.

Variable	FRE (*n* = 28)	ARE (*n* = 5)	IRE/NRE (*n* = 15)	*p* Value
BCS (1–9)	5 (2–6)	3 (2–5)	4 (1–7)	0.175
CCECAI	3 (1–12)	6 (5–13)	6 (2–19)	*0.003* *
CRP (0–1 mg/dL)	0.6 (0.0–12.4)	1.0 (0.9–2.3)	1.0 (0.0–10.0)	0.340
Folate (6.5–11.5 µg/L)	8.9 (2.2–24.0)	7.9 (2.6–11.0)	7.7 (1.0–24.0)	0.891
Cobalamin (250–730 ng/L)	397 (150–824)	284 (242–575)	307 (150–994)	0.356

* Statistical significance between FRE and IRE/NRE groups.

**Table 3:** Reference intervals reported as interquartile range with number and percentage (in brackets) of dogs affected by chronic enteropathy with values outside the interval.

Variable	Healthy Dogs (*n* = 68)	Chronic Enteropathy (*n* = 48)	
	Interquartile (IQ) Range	Number (Percentage) of Dogs with Values above the IQ Range	Number (Percentage) of Dogs with Values below the IQ Range
Palmitic Acid	13.03–17.43	0 (0%)	42 (87.5%)
Stearic Acid	18.95–21.81	41 (85.4%)	0 (0%)
**Saturated Fatty Acids**	33.22–37.44	4 (8.3%)	11 (22.9%)
Palmitoleic Acid	0.19–0.33	24 (50%)	11 (22.9%)
Oleic Acid	8.50–10.16	14 (29.1%)	8 (16.6%)
Vaccenic Acid	1.77–2.28	18 (37.5%)	11 (22.9%)
**Monounsaturated Fatty Acids**	10.43–12.72	17 (35.4%)	5 (10.4%)
Linoleic Acid	13.15–16.22	6 (12.5%)	25 (52.0%)
Dihomo-gamma-linolenic Acid	1.06–1.57	28 (58.3%)	4 (8.3%)
Arachidonic Acid	31.43–37.64	10 (20.8%)	2 (4.1%)
ω-6 Polyunsaturated Fatty Acids	47.27–53.20	12 (25.0%)	7 (14.5%)
EPA	0.46–0.89	22 (45.8%)	11 (22.9%)
DHA	0.70–1.68	14 (29.1%)	4 (8.3%)
ω-3 Polyunsaturated Fatty Acids	1.20–2.40	21 (43.7%)	5 (10.4%)
**Polyunsaturated Fatty Acids**	49.97–55.29	12 (25.0%)	7 (14.5%)
SFA/MUFA	2.83–3.28	8 (16.6%)	25 (52.0%)
ω-6/ω-3	19.60–40.18	4 (8.3%)	14 (29.1%)
PUFA Balance	2.42–4.85	14 (29.1%)	4 (8.3%)
Unsaturation Index	186.8–207.2	14 (29.1%)	4 (8.3%)
Peroxidation Index	160.7–181.9	17 (35.4%)	5 (10.4%)
Elongase-6 activity	0.56–0.71	43 (89.5%)	0 (0.0%)
Delta-6 desaturase activity	0.07–0.11	32 (66.6%)	3 (6.2%)
Delta-5 desaturase activity	21.09–30.51	7 (14.5%)	18 (37.5%)
Delta-9 desaturase activity	0.40–0.51	4 (8.3%)	20 (41.6%)

Saturated Fatty Acids (Total SFAs) = % C16:0 + % C18:0; Monounsaturated Fatty Acids (Total MUFAs) = % C16:1 + % 9c, C18:1 + % 11c, C18:1; ω-3 Polyunsaturated Fatty Acids = %EPA + %DHA; ω-6 Polyunsaturated Fatty Acids = %LA + %DGLA + %ARA; Polyunsaturated Fatty Acids (PUFAs) = %LA + %DGLA + %ARA + %EPA + %DHA; SFA/MUFA = (% C16:0 + % C18:0)/(% C16:1 + % 9c, C18:1 + % 11c, C18:1); ω-6/ω-3 ratio = (%LA + %DGLA + %ARA)/ (%EPA + %DHA); PUFA balance = [(%EPA + %DHA)/Total PUFAs] × 100; Unsaturation index (UI) = (%MUFA × 1) + (%LA × 2) + (%DGLA × 3) + (%ARA × 4) + (%EPA × 5) + (%DHA × 6); Peroxidation index (PI) = (%MUFA × 0.025) + (%LA × 1) + (%DGLA × 2) + (%ARA × 4) + (%EPA × 6) + (%DHA × 8); Elongase-6 activity (EI) = C18:0/C16:0; Delta-9 desaturase activity (D9DI) = 9c, C18:1/C18:0; Delta-6 desaturase activity (D6DI) = C20:3/C18:2; andDelta-5 desaturase (D5DI) = C20:4/C20:3.

**Table 4:** Spearman’s rank correlation coefficient (Spearman’s rho) for fatty acids, fatty acids families, homeostasis, and enzyme indexes with the clinical and clinicopathological characteristics of dogs affected by chronic enteropathy. EPA, eicosapentaenoic acid; DHA, docosahexaenoic acid; SFA, saturated fatty acid; MUFA, monounsaturated fatty acid; PUFA, polyunsaturated fatty acid; BCS, Body Condition Score; CCECAI, Canine Chronic Enteropathy Clinical Activity Index; and CRP, C-reactive protein.

Fatty Acids	BCS	CCECAI	CRP	Folate	Cobalamin
Palmitic Acid	0.029	0.051	0.313	−0.119	−0.128
Stearic Acid	0.092	0.222	−0.018	−0.138	−0.035
Palmitoleic Acid	0.095	0.031	0.075	0.107	−0.020
Oleic Acid	−0.031	0.245	0.183	−0.355 *	−0.148
Vaccenic Acid	−0.044	0.223	0.222	−0.347 *	−0.362 *
Linoleic Acid	−0.165	−0.144	−0.013	0.177	0.125
Dihomo-gamma-linolenic Acid	−0.103	0.021	−0.067	−0.241	−0.206
Arachidonic Acid	−0.004	−0.186	−0.207	0.309	0.166
EPA	0.06	−0.241	−0.194	0.142	0.080
DHA	0.125	−0.011	−0.101	0.147	−0.108
**Fatty Acid Families**					
Total SFAs	0.126	0.195	0.193	−0.234	−0.013
Total MUFAs	−0.022	0.222	0.146	−0.365 *	−0.204
ω-6 Polyunsaturated Fatty Acids	−0.097	−0.300 *	−0.229	0.330 *	0.181
ω-3 Polyunsaturated Fatty Acids	0.134	−0.185	−0.079	0.171	−0.032
Total PUFAs	−0.077	−0.283	−0.276	0.369 *	0.128
**Homeostasis Indexes**					
SFA/MUFA	0.097	−0.116	−0.0114	0.249	0.116
ω-6/ω-3	−0.125	0.142	0.0578	−0.112	0.069
PUFA Balance	0.125	−0.142	−0.0578	0.112	−0.069
Unsaturation Index	−0.01	−0.250	−0.275	0.317	0.106
Peroxidation Index	0.064	−0.250	−0.232	0.319	0.087
**Enzyme indexes**					
Elongase-6 activity	−0.04	0.081	−0.147	−0.099	0.021
Delta-6 desaturase activity	0.001	0.107	−0.074	−0.340 *	−0.358 *
Delta-5 desaturase activity	0.087	−0.042	0.043	0.327 *	0.235
Delta-9 desaturase activity	−0.059	0.026	0.114	−0.128	−0.034

* Statistical significance at 0.05 level Saturated Fatty Acids (Total SFAs) = % C16:0 + % C18:0; Monounsaturated Fatty Acids (Total MUFAs) = % C16:1 + % 9c, C18:1 + % 11c, C18:1; ω-3 Polyunsaturated Fatty Acids = %EPA + %DHA; ω-6 Polyunsaturated Fatty Acids = %LA + %DGLA + %ARA; Polyunsaturated Fatty Acids (PUFAs) = %LA + %DGLA + %ARA + %EPA + %DHA; SFA/MUFA = (% C16:0 + % C18:0)/(% C16:1 + % 9c, C18:1 + % 11c, C18:1); ω-6/ω-3 ratio = (%LA + %DGLA + %ARA)/ (%EPA + %DHA); PUFA balance = [(%EPA + %DHA)/Total PUFAs] × 100; Unsaturation index (UI) = (%MUFA × 1) + (%LA × 2) + (%DGLA × 3) + (%ARA × 4) + (%EPA × 5) + (%DHA × 6); Peroxidation index (PI) = (%MUFA × 0.025) + (%LA × 1) + (%DGLA × 2) + (%ARA × 4) + (%EPA × 6) + (%DHA × 8); Elongase-6 activity (EI) = C18:0/C16:0; Delta-9 desaturase activity (D9DI) = 9c, C18:1/C18:0; Delta-6 desaturase activity (D6DI) = C20:3/C18:2; and Delta-5 desaturase (D5DI) = C20:4/C20:3.

## Data Availability

Data will be available upon request.

## Supplementary Materials

The following are available online at https://www.mdpi.com/article/10.3390/ani11092604/s1, Table S1. Breed, sex, age and bodyweight of the recruited dogs affected by chronic enteropathy (n = 48), Table S2. Median values with minimum and maximum in brackets of fatty acids, homeostasis in-dexes and enzyme activity indexes of healthy dogs and dogs affected by chronic enteropathy. Fatty acids are evaluated as fatty acid methyl esters (FAME) after membrane isolation, lipid ex-traction and derivatization, Table S3. Median values with minimum and maximum in brackets of fatty acids, homeostasis indexes and enzyme activity indexes of CE dogs, divided in groups affected by food-responsive enteropathy (FRE), antibiotic-responsive enteropathy (ARE), immunosuppressant-responsive enteropathy (IRE) and non-responsive enteropathy (NRE), Table S4. Median values with minimum and maximum in brackets of fatty acids, homeostasis indexes and enzyme activity indexes of dogs affected by chronic enteropathies without and with loss of protein across the intestine. Fatty acids are obtained as fatty acid methyl esters (FAME) after membrane isolation, lipid extraction and derivatization, Table S5. Median values with minimum and maximum in brackets of fatty acids, homeostasis indexes and enzyme activity indexes of that responded to therapeutic trials and dogs that poorly or not responded (NRE), Figure S1. Steps of the laboratory procedure from the blood sample in EDTA to the fatty acid-based RBC membrane profile, Figure S2. Calibration curves of the 10 fatty acids at high (0.5–5 mM) and low (from 0.001 mM to 0.5 mM) concentration ranges, chosen as representatives of the SFA, MUFA and PUFA families present in the erythrocyte membrane phospholipids, Figure S3. Representative GC chromatogram of the FAME obtained from dog erythrocyte membrane phospholipids after work-up, as described in the main text, Figure S4. Spearman rank correlation between: (A) C-reactive protein and palmitic acid, (B) the Canine Chronic Enteropathy Clinical Activity Index and ω-6 polyunsaturated fatty acids,

## References

[1] Dandrieux J.R.S. (2016). Inflammatory bowel disease versus chronic enteropathy in dogs: Are they one and the same?. J. Small Anim. Pr..

[2] Washabau R.J., Day M.J., Willard M.D., Hall E., Jergens A.E., Mansell J., Minami T., Bilzer T.W., The WSAVA International Gastrointestinal Standardization Group (2010). Endoscopic, Biopsy, and Histopathologic Guidelines for the Evaluation of Gastrointestinal Inflammation in Companion Animals. J. Vet. Intern. Med..

[3] Allenspach K., Wieland B., Gröne A., Gaschen F. (2007). Chronic Enteropathies in Dogs: Evaluation of Risk Factors for Negative Outcome. J. Vet. Intern. Med..

[4] Craven M., Simpson J.W., Ridyard A.E., Chandler M.L. (2004). Canine inflammatory bowel disease: Retrospective analysis of diagnosis and outcome in 80 cases (1995–2002). J. Small Anim. Pr..

[5] Nakashima K., Hiyoshi S., Ohno K., Uchida K., Goto-Koshino Y., Maeda S., Mizutani N., Takeuchi A., Tsujimoto H. (2015). Prognostic factors in dogs with protein-losing enteropathy. Vet. J..

[6] German A., Hall E.J., Day M.J. (2003). Chronic intestinal inflammation and intestinal disease in dogs. J. Vet. Intern. Med..

[7] Hall E.J. (1994). Gastrointestinal aspects of food allergy: A review. J. Small Anim. Pr..

[8] Suchodolski J.S., Markel M.E., Garcia-Mazcorro J., Unterer S., Heilmann R.M., Dowd S., Kachroo P., Ivanov I., Minamoto Y., Dillman E.M. (2012). The Fecal Microbiome in Dogs with Acute Diarrhea and Idiopathic Inflammatory Bowel Disease. PLoS ONE.

[9] Scaioli E., Liverani E., Belluzzi A. (2017). The Imbalance between n-6/n-3 Polyunsaturated Fatty Acids and Inflammatory Bowel Disease: A Comprehensive Review and Future Therapeutic Perspectives. Int. J. Mol. Sci..

[10] Dumusc S., Ontsouka E., Schnyder M., Hartnack S., Albrecht C., Bruckmaier R., Burgener I. (2014). Cyclooxygenase-2 and 5-Lipoxygenase in Dogs with Chronic Enteropathies. J. Vet. Intern. Med..

[11] Michalak A., Mosińska P., Fichna J. (2016). Polyunsaturated Fatty Acids and Their Derivatives: Therapeutic Value for Inflammatory, Functional Gastrointestinal Disorders, and Colorectal Cancer. Front. Pharmacol..

[12] de Carvalho M.J.C.R., Caramujo M.J. (2018). The Various Roles of Fatty Acids. Molecules.

[13] Nicolson G.L., Ash M. (2014). Lipid Replacement Therapy: A natural medicine approach to replacing damaged lipids in cellular membranes and organelles and restoring function. Biochim. Biophys. Acta (BBA) Biomembr..

[14] Ferreri C., Chatgilialoglu C. (2015). Nutrilipidomics. Membrane Lipidomics for Personalized Health.

[15] Ferreri C., Masi A., Sansone A., Giacometti G., LaRocca A.V., Menounou G., Scanferlato R., Tortorella S., Rota D., Conti M. (2016). Fatty Acids in Membranes as Homeostatic, Metabolic and Nutritional Biomarkers: Recent Advancements in Analytics and Diagnostics. Diagnostics.

[16] Ferreri C., Chatgilialoglu C. (2012). Role of fatty acid-based functional lipidomics in the development of molecular diagnostic tools. Expert Rev. Mol. Diagn..

[17] Hu C., van der Heijden R., Wang M., van der Greef J., Hankemeier T., Xu G. (2009). Analytical strategies in lipidomics and applications in disease biomarker discovery. J. Chromatogr. B.

[18] Alhouayek M., Ameraoui H., Muccioli G.G. (2021). Bioactive lipids in inflammatory bowel diseases–From pathophysiological alterations to therapeutic opportunities. Biochim. Biophys. Acta (BBA) Mol. Cell Biol. Lipids.

[19] Barnig C., Bezema T., Calder P.C., Charloux A., Frossard N., Garssen J., Haworth O., Dilevskaya K., Levi-Schaffer F., Lonsdorfer E. (2019). Activation of Resolution Pathways to Prevent and Fight Chronic Inflammation: Lessons from Asthma and Inflammatory Bowel Disease. Front. Immunol..

[20] Buhner S., Nagel E., Korber J., Vogelsang H., Linn T., Pichlmayr R. (1994). Ileal and colonic fatty acid profiles in patients with active Crohn’s disease. Gut.

[21] Fernández–Bañares F., Esteve–Comas M., Mañé J., Navarro E., Bertrán X., Cabré E., Bartolí R., Boix J., Pastor C., Gassull M.A. (1997). Changes in Mucosal Fatty Acid Profile in Inflammatorybowel Disease and in Experimental Colitis: A Common Response to Bowel Inflammation. Clin. Nutr..

[22] Hwang D.H., Kim J., Lee J.Y. (2016). Mechanisms for the activation of Toll-like receptor 2/4 by saturated fatty acids and inhibition by docosahexaenoic acid. Eur. J. Pharmacol..

[23] Kalenyak K., Heilmann R.M., Van De Lest C.H.A., Brouwers J.F., Burgener I.A. (2019). Comparison of the systemic phospholipid profile in dogs diagnosed with idiopathic inflammatory bowel disease or food-responsive diarrhea before and after treatment. PLoS ONE.

[24] Ambrosini Y.M., Neuber S., Borcherding D., Seo Y.-J., Segarra S., Glanemann B., Garden O.A., Müller U., Adam M.G., Dang V. (2020). Treatment with Hydrolyzed Diet Supplemented with Prebiotics and Glycosaminoglycans Alters Lipid Metabolism in Canine Inflammatory Bowel Disease. Front. Vet. Sci..

[25] Sender R., Fuchs S., Milo R. (2016). Revised Estimates for the Number of Human and Bacteria Cells in the Body. PLoS Biol..

[26] Acar N., Berdeaux O., Grégoire S., Cabaret S., Martine L., Gain P., Thuret G., Creuzot-Garcher C.P., Bron A.M., Bretillon L. (2012). Lipid Composition of the Human Eye: Are Red Blood Cells a Good Mirror of Retinal and Optic Nerve Fatty Acids?. PLoS ONE.

[27] Dushianthan A., Cusack R., Koster G., Grocott M.P.W., Postle A.D. (2019). Insight into erythrocyte phospholipid molecular flux in healthy humans and in patients with acute respiratory distress syndrome. PLoS ONE.

[28] Arab L. (2003). Biomarkers of Fat and Fatty Acid Intake. J. Nutr..

[29] Barrows B.R., Parks E.J. (2006). Contributions of Different Fatty Acid Sources to Very Low-Density Lipoprotein-Triacylglycerol in the Fasted and Fed States. J. Clin. Endocrinol. Metab..

[30] Timlin M.T., Parks E.J. (2005). Temporal pattern of de novo lipogenesis in the postprandial state in healthy men. Am. J. Clin. Nutr..

[31] Prasinou P., Crisi P.E., Chatgilialoglu C., Di Tommaso M., Sansone A., Gramenzi A., Belà B., De Santis F., Boari A., Ferreri C. (2020). The Erythrocyte Membrane Lipidome of Healthy Dogs: Creating a Benchmark of Fatty Acid Distribution and Interval Values. Front. Vet. Sci..

[32] Heilmann R.M., Berghoff N., Mansell J., Grützner N., Parnell N.K., Gurtner C., Suchodolski J.S., Steiner J.M. (2018). Association of fecal calprotectin concentrations with disease severity, response to treatment, and other biomarkers in dogs with chronic inflammatory enteropathies. J. Vet. Intern. Med..

[33] Fuchs B., Süß R., Teuber K., Eibisch M., Schiller J. (2011). Lipid analysis by thin-layer chromatography—A review of the current state. J. Chromatogr. A.

[34] Sansone A., Tolika E., Louka M., Sunda V., Deplano S., Melchiorre M., Anagnostopoulos D., Chatgilialoglu C., Formisano C., Di Micco R. (2016). Hexadecenoic Fatty Acid Isomers in Human Blood Lipids and Their Relevance for the Interpretation of Lipidomic Profiles. PLoS ONE.

[35] Do H.J., Chung H.K., Moon J., Shin M.J. (2011). Relationship between the Estimates of Desaturase Activities and Cardiometabolic Phenotypes in Koreans. J Clin. Biochem. Nutr..

[36] Scanferlato R., Bortolotti M., Sansone A., Chatgilialoglu C., Polito L., De Spirito M., Maulucci G., Bolognesi A., Ferreri C. (2019). Hexadecenoic Fatty Acid Positional Isomers and De Novo PUFA Synthesis in Colon Cancer Cells. Int. J. Mol. Sci..

[37] Uchiyama K., Odahara S., Nakamura M., Koido S., Katahira K., Shiraishi H., Ohkusa T., Fujise K., Tajiri H. (2013). The Fatty Acid Profile of the Erythrocyte Membrane in Initial-Onset Inflammatory Bowel Disease Patients. Dig. Dis. Sci..

[38] Riezzo G., Ferreri C., Orlando A., Martulli M., D’Attoma B., Russo F. (2014). Lipidomic analysis of fatty acids in erythrocytes of coeliac patients before and after a gluten-free diet intervention: A comparison with healthy subjects. Br. J. Nutr..

[39] Ueda Y., Kawakami Y., Kunii D., Okada H., Azuma M., Le D.S.N., Yamamoto S. (2008). Elevated concentrations of linoleic acid in erythrocyte membrane phospholipids in patients with inflammatory bowel disease. Nutr. Res..

[40] Abbott S.K., Else P., Atkins T.A., Hulbert A. (2012). Fatty acid composition of membrane bilayers: Importance of diet polyunsaturated fat balance. Biochim. Biophys. Acta (BBA) Biomembr..

[41] Baur L.A., O’Connor J., Pan D.A., Wu B., O’Connor M.J., Storlien L.H. (2000). Relationships between the fatty acid composition of muscle and erythrocyte membrane phospholipid in young children and the effect of type of infant feeding. Lipids.

[42] Makrides M., Neumann M.A., Byard R., Simmer K., Gibson R. (1994). Fatty acid composition of brain, retina, and erythrocytes in breast- and formula-fed infants. Am. J. Clin. Nutr..

[43] Bueno-Hernández N., Dominguez-López A., Barreto-Zuñiga R., Sánchez-Muñoz F., Yamamoto-Furusho J.K. (2011). Quantification of low expressed SCD1 gene in colonic mucosa from patients with active ulcerative colitis. Inflamm. Bowel Dis..

[44] Chen C., Shah Y.M., Morimura K., Krausz K.W., Miyazaki M., Richardson T.A., Morgan E., Ntambi J.M., Idle J., Gonzalez F.J. (2008). Metabolomics Reveals that Hepatic Stearoyl-CoA Desaturase 1 Downregulation Exacerbates Inflammation and Acute Colitis. Cell Metab..

[45] Wang R., Gu X., Dai W., Ye J., Lu F., Chai Y., Fan G., Gonzalez F.J., Duan G., Qi Y. (2016). A lipidomics investigation into the intervention of celastrol in experimental colitis. Mol. BioSyst..

[46] Jezernik G., Potočnik U. (2018). Comprehensive genetic study of fatty acids helps explain the role of noncoding inflammatory bowel disease associated SNPs and fatty acid metabolism in disease pathogenesis. Prostagland. Leukot. Essent. Fat. Acids.

[47] Nicholas D., Zhang K., Hung C., Glasgow S., Aruni A.W., Unternaehrer J., Payne K., Langridge W.H.R., De Leon M. (2017). Palmitic acid is a toll-like receptor 4 ligand that induces human dendritic cell secretion of IL-1β. PLoS ONE.

[48] Schnyder M., Oevermann A., Doherr M.G., Luckschander N., Zurbriggen A., Burgener I.A. (2018). Dysregulation of Toll-Like Receptors in Dogs with Chronic Enteropathies. J. Inflamm. Bowel Dis. Disord..

[49] Burgener I., König A., Allenspach K., Sauter S., Boisclair J., Doherr M., Jungi T. (2008). Upregulation of Toll-Like Receptors in Chronic Enteropathies in Dogs. J. Vet. Intern. Med..

[50] Gugliandolo E., Peritore A.F., Piras C., Cuzzocrea S., Crupi R. (2020). Palmitoylethanolamide and Related ALIAmides: Prohomeostatic Lipid Compounds for Animal Health and Wellbeing. Vet. Sci..

[51] Febo E., Crisi P.E., Oddi S., Pietra M., Galiazzo G., Piscitelli F., Gramenzi A., Di Prinzio R., Di Tommaso M., Bernabò N. (2021). Circulating Endocannabinoids as Diagnostic Markers of Canine Chronic Enteropathies: A Pilot Study. Front. Vet. Sci..

[52] Wang Y., Balvers M.G., Hendriks H.F., Wilpshaar T., Van Heek T., Witkamp R.F., Meijerink J. (2017). Docosahexaenoyl serotonin emerges as most potent inhibitor of IL-17 and CCL-20 released by blood mononuclear cells from a series of N -acyl serotonins identified in human intestinal tissue. Biochim. Biophys. Acta (BBA) Mol. Cell Biol. Lipids.

[53] Ito Z., Uchiyama K., Takami S., Koido S., Odahara S., Kubota T., Ohkusa T. (2015). Utility of Fatty Acid Profile of Erythrocyte Membrane in Identifying Diagnostic Markers of Crohn’s Disease. Am. J. Gastroenterol..

[54] Solakivi T., Kaukinen K., Kunnas T., Lehtimäki T., Mäki M., Nikkari S.T. (2009). Serum fatty acid profile in celiac disease patients before and after a gluten-free diet. Scand. J. Gastroenterol..

[55] Chambrier C., Garcia I., Bannier E., Gerard-Boncompain M., Bouletreau P. (2002). Specific changes in n -6 fatty acid metabolism in patients with chronic intestinal failure. Clin. Nutr..

[56] Steel D.M., Ryd W., Ascher H., Strandvik B. (2006). Abnormal Fatty Acid Pattern in Intestinal Mucosa of Children with Celiac Disease Is Not Reflected in Serum Phospholipids. J. Pediatr. Gastroenterol. Nutr..

[57] Ananthakrishnan A.N., Khalili H., Song M., Higuchi L.M., Lochhead P., Richter J.M., Chan A.T. (2017). Genetic Polymorphisms in Fatty Acid Metabolism Modify the Association Between Dietary n3. Inflamm. Bowel Dis..

[58] Costea I., Mack D.R., Lemaitre R.N., Israel D., Marcil V., Ahmad A., Amre D.K. (2014). Interactions Between the Dietary Polyunsaturated Fatty Acid Ratio and Genetic Factors Determine Susceptibility to Pediatric Crohn’s Disease. Gastroenterology.

[59] Costea I., Mack D.R., Israel D., Morgan K., Krupoves A., Seidman E., Deslandres C., Lambrette P., Grimard G., Levy E. (2010). Genes Involved in the Metabolism of Poly-Unsaturated Fatty-Acids (PUFA) and Risk for Crohn’s Disease in Children & Young Adults. PLoS ONE.

[60] Marion-Letellier R., Savoye G., Beck P.L., Panaccione R., Ghosh S. (2013). Polyunsaturated Fatty Acids in Inflammatory Bowel Diseases. Inflamm. Bowel Dis..

[61] Trebble T.M., Arden N.K., Wootton S., Calder P., Mullee M.A., Fine D., Stroud M.A. (2004). Fish oil and antioxidants alter the composition and function of circulating mononuclear cells in Crohn disease. Am. J. Clin. Nutr..

[62] Esteve M., Ramírez M., Fernández-Bañares F., Lacruz A.A., Gil A., Cabre E., Gonzalez-Huix F., Moreno J., Humbert P., Guilera M. (1992). Plasma polyunsaturated fatty acid pattern in active inflammatory bowel disease. Gut.

[63] Esteve M., Núñez M.C., Fernández-Bañares F., Abad-Lacruz A., Gil A., Cabre E., Gonzalez-Huix F., Bertrán X., Gassull M. (1993). Àngel Abnormal plasma polyunsaturated fatty acid pattern in non-active inflammatory bowel disease. Gut.

[64] Daniluk U., Daniluk J., Kucharski R., Kowalczyk T., Pietrowska K., Samczuk P., Filimoniuk A., Kretowski A., Lebensztejn D., Ciborowski M. (2019). Untargeted Metabolomics and Inflammatory Markers Profiling in Children With Crohn’s Disease and Ulcerative Colitis—A Preliminary Study. Inflamm. Bowel Dis..

[65] Scoville E., Allaman M.M., Adams D.W., Motley A.K., Peyton S.C., Ferguson S.L., Horst S.N., Williams C., Beaulieu D.B., Schwartz D.A. (2019). Serum Polyunsaturated Fatty Acids Correlate with Serum Cytokines and Clinical Disease Activity in Crohn’s Disease. Sci. Rep..

[66] Wiese D.M., Horst S.N., Brown C.T., Allaman M.M., Hodges M.E., Slaughter J., Druce J.P., Beaulieu D.B., Schwartz D.A., Wilson K.T. (2016). Serum Fatty Acids Are Correlated with Inflammatory Cytokines in Ulcerative Colitis. PLoS ONE.

[67] Sergeant S., Rahbar E., Chilton F.H. (2016). Gamma-linolenic acid, Dihommo-gamma linolenic, Eicosanoids and Inflammatory Processes. Eur. J. Pharmacol..

[68] Burr G.O., Burr M.M. (1929). A new deficiency disease produced by the rigid exclusion of fat from the diet. J. Biol. Chem..

[69] Revskij D., Haubold S., Viergutz T., Kröger-Koch C., Tuchscherer A., Kienberger H., Rychlik M., Tröscher A., Hammon H.M., Schuberth H.-J. (2019). Dietary Fatty Acids Affect Red Blood Cell Membrane Composition and Red Blood Cell ATP Release in Dairy Cows. Int. J. Mol. Sci..

[70] Perona J.S. (2017). Membrane lipid alterations in the metabolic syndrome and the role of dietary oils. Biochim. Biophys. Acta (BBA) Biomembr..

[71] Symons J., Cho K.-J., Chang J.T., Du G., Waxham M.N., Hancock J.F., Levental I., Levental K.R. (2021). Lipidomic atlas of mammalian cell membranes reveals hierarchical variation induced by culture conditions, subcellular membranes, and cell lineages. Soft Matter.

[72] Pamplona R., Portero–Otín M., Riba D., Ruiz C., Prat J., Bellmunt M.J., Barja G. (1998). Mitochondrial Membrane Peroxidizability Index Is Inversely Related to Maximum Life Span in Mammals. J. Lipid Res..

[73] Rubio C., Martinez-Subiela S., Ruiz J.H., Tvarijonaviciute A., Cerón J., Allenspach K. (2017). Serum biomarkers of oxidative stress in dogs with idiopathic inflammatory bowel disease. Vet. J..

[74] Tan M., Ye J., Zhou Z., Ke X., Yu X., Huang K. (2020). Fatty Acid Metabolism in Immune Cells: A Bioinformatics Analysis of Genes Involved in Ulcerative Colitis. DNA Cell Biol..

[75] Robichaud P.-P., Munganyiki J.E., Boilard E., Surette M.E. (2018). Polyunsaturated fatty acid elongation and desaturation in activated human T-cells: ELOVL5 is the key elongase. J. Lipid Res..

[76] Das U.N. (2008). Folic acid and polyunsaturated fatty acids improve cognitive function and prevent depression, dementia, and Alzheimer’s disease—But how and why?. Prostagland. Leukot. Essent. Fat. Acids.

[77] Ued F.V., Mathias M.G., Toffano R.B.D., Barros T.T., Almada M.O.R.V., Salomão R.G., Coelho-Landell C.A., Hillesheim E., Camarneiro J.M., Camelo-Junior J.S. (2019). Vitamin B2 and Folate Concentrations are Associated with ARA, EPA and DHA Fatty Acids in Red Blood Cells of Brazilian Children and Adolescents. Nutrition.

[78] Umhau J.C., Dauphinais K.M., Patel S.H., Nahrwold D.A., Hibbeln J.R., Rawlings R.R., George D.T. (2005). The relationship between folate and docosahexaenoic acid in men. Eur. J. Clin. Nutr..

[79] Selhub J. (2002). Folate, vitamin B12 and vitamin B6 and one carbon metabolism. J. Nutr. Heal. Aging.

[80] Ferreri C., Grabovskiy S.A., Aoun M., Melchiorre M., Kabal’Nova N., Feillet-Coudray C., Fouret G., Coudray C., Chatgilialoglu C. (2012). Trans Fatty Acids: Chemical Synthesis of Eicosapentaenoic Acid Isomers and Detection in Rats Fed a Deodorized Fish Oil Diet. Chem. Res. Toxicol..

[81] Menounou G., Giacometti G., Scanferlato R., Dambruoso P., Sansone A., Tueros I., Amézaga J., Chatgilialoglu C., Ferreri C. (2018). Trans Lipid Library: Synthesis of Docosahexaenoic Acid (DHA) Monotrans Isomers and Regioisomer Identification in DHA-Containing Supplements. Chem. Res. Toxicol..

