# Supplementary material for: The Fatty Acid-Based Erythrocyte Membrane Lipidome in Dogs with Chronic Enteropathy
Source: Animals (Basel). 2021 Sep 5;11(9):2604. doi: 10.3390/ani11092604 (PMC8469057; doi:10.3390/ani11092604)
Supplement: Supplementary file 1 [file animals-11-02604-s001.zip › animals-1268852-supplementary.pdf]

animals

Supplementary Material

The fatty acid-based erythrocyte membrane lipidome in dogs with chronic enteropathy

Paolo E. Crisi[1], Alessia Luciani[1†], Morena Di Tommaso[1†], Paraskevi Prasinou[1], Francesca De Santis[1], Chryssostomos Chatgilialoglu[2], Marco Pietra[3], Fabio Procoli[4], Anna Sansone[2], Maria Veronica Giordano[1], Alessandro Gramenzi[1*], Carla Ferreri[2*] and Andrea Boari[1]

1. Supplementary tables

Supplementary Table S1. Breed, sex, age and bodyweight of the recruited dogs affected by chronic enteropathy (n=48).

N	Breed	Sex	Age (Months)	BW (Kg)	Diagnosis
1	Miniature Poodle	M	84	5,3	IRE (PLE)
2	Labrador Retriever	M	12	35,0	FRE
3	French Bouledogue	M	15	10,3	FRE
4	Staffordshire Terrier	M	108	23,0	FRE
5	Border collie	M	72	25,2	FRE
6	English Setter	M	48	16,0	FRE
7	American Staffordshire Terrier	M	47	23,1	FRE
8	French Bouledogue	Fs	22	10,6	IRE
9	Mixed-breed	F	42	18,5	FRE
10	Jack Russell Terrier	F	10	4,4	FRE
11	English Bull terrier	M	35	26,0	ARE
12	Pitbull	M	66	22,0	IRE (PLE)
13	Dogo Argentino	M	96	35,5	IRE
14	Boxer	M	87	17,0	NRE (PLE)
15	French Bouledogue	M	13	7,1	FRE
16	German Shepherd	Fs	41	25,0	FRE
17	Miniature Poodle	M	83	6,0	FRE
18	Belgian Shepherd Dog (Malinois)	M	24	22,0	FRE
19	Maltese	Fs	114	4,0	FRE
20	German Shepherd	M	14	28,0	IRE
21	Mixed-breed	M	37	36,3	ARE
22	Mixed-breed	F	32	12,5	ARE
23	Labrador Retriever	Fs	76	28,2	IRE
24	Dobermann	M	11	30,5	ARE
25	Boxer	M	12	32,3	FRE
26	Dachshund	F	96	4,0	FRE
27	Mixed-breed	F	96	25,0	FRE
28	Maltese	F	36	4,0	FRE
29	Golden Retriever	M	24	31,0	FRE
30	Bolognese	M	104	4,6	ARE (PLE)
31	Italian Bloodhound	F	48	15,0	IRE (PLE)
32	German Shepherd	M	14	34,5	FRE
33	Bull terrier	F	33	15,5	FRE

34	Labrador Retriever	M	10	31,0	IRE
35	Portuguese Sheepdog (Cao da Serra de Aires)	M	131	12,6	FRE
36	Jack Russell Terrier	Fs	48	5,5	IRE
37	Labrador Retriever	F	24	29,7	NRE (PLE)
38	Belgian Shepherd Dog (Groenendael)	Mc	45	28,0	FRE
39	Basset Hound	M	55	23,0	FRE
40	Golden Retriever	Fs	48	28,3	FRE
41	Mixed-breed	F	84	7,3	IRE
42	Mixed-breed	M	96	17,0	NRE (PLE)
43	Miniature Poodle	M	144	6,7	IRE (PLE)
44	English Setter	M	4	21,5	IRE
45	Mixed-breed	Fs	96	22,5	FRE
46	Kurzhaar	M	72	20,2	FRE (PLE)
47	Pitbull	M	12	30,0	FRE
48	Boxer	M	18	30,0	FRE

M: male; F: female; Mn: neutered male; Fs: spayed female; FRE: food-responsive enteropathy; ARE: antibiotic-responsive enteropathy; IRE: immunosuppressive-responsive enteropathy; NRE: non-responsive enteropathy; PLE: protein-losing enteropathy.

Supplementary Table S2. Median values with minimum and maximum in brackets of fatty acids, homeostasis indexes and enzyme activity indexes of healthy dogs and dogs affected by chronic enteropathy. Fatty acids are evaluated as fatty acid methyl esters (FAME) after membrane isolation, lipid extraction and derivatization. The values are expressed as percentage of the found quantities (calculated as μg/mL) from the gas chromatographic analysis, using calibration and quantitation protocols and standard reference compounds for each FAME, as described in Materials and Methods. The GC peak areas of the 10 fatty acids cohort corresponds to ca. 97% of the total peak areas of the chromatogram. Italic values denote statistical significance.

Variable	Healthy Dogs (n = 68)	Chronic Enteropathy (n = 48)	P value
Palmitic Acid	15.38 (8.25-25.82)	11.01 (4.91-13.94)	*<0.0001*
Stearic Acid	20.22 (15.61-27.31)	23.29 (20.15-28.14)	*<0.0001*
**Saturated Fatty Acids**	35.54 (27.88-53.13)	34.55 (29.26-41.63)	*0.026*
Palmitoleic Acid	0.26 (0.08-1.40)	0.30 (0.05-2.23)	0.066
Oleic Acid	9.18 (6.97-24.49)	9.48 (7.49-14.68)	0.232
Vaccenic Acid	1.95 (1.21-2.65)	2.08 (0.07-3.35)	0.109
**Monounsaturated Fatty Acids**	11.70 (8.77-26.38)	12.17 (8.44-16.62)	0.080
Linoleic Acid	14.52 (9.26-21.08)	13.06 (7.39-18.98)	*0.0008*
Dihomo-gamma-linolenic Acid	1.27 (0.46-2.30)	1.63 (0.14-3.35)	*0.0001*
Arachidonic Acid	35.04 (13.44-45.87)	35.89 (29.70-43.75)	0.11
ω-6 Polyunsaturated Fatty Acids	50.97 (31.32-60-76)	50.93 (43.22-57.10)	0.828
EPA	0.67 (0.25-2.01)	0.85 (0.11-2.00)	*0.029*
DHA	0.99 (0.16-2.61)	1.23 (0.42-3.63)	*0.031*
ω-3 Polyunsaturated Fatty Acids	1.75 (0.52-4.03)	2.19 (0.71-4.71)	*0.013*
**Polyunsaturated Fatty Acids**	53.00 (32.98-61.82)	53.00 (44.42-59.94)	0.545
SFA/MUFA	3.03 (1.42-3.89)	2.80 (2.04-3.88)	*0.002*
ω-6/ω-3	29.04 (12.51-100.3)	22.44 (10.12-72.68)	*0.045*
PUFA Balance	3.33 (0.99-7.40)	4.27 (1.35-8.99)	*0.045*

Unsaturation Index	194.6 (121.7-233.5)	200.2 (173.9-221.8)	*0.018*
Peroxidation Index	171.0 (84.83-213.7)	178.3 (149.8-203.0)	*0.020*
Elongase-6 activity	0.61 (0.42-1.20)	0.87 (0.67-1.65)	*<0.0001*
Delta-6 desaturase activity	0.09 (0.02-0.18)	0.12 (0.01-0.27)	*<0.0001*
Delta-5 desaturase activity	25.74 (14.36-65.31)	22.54 (10.48-257.20)	*0.0057*
Delta-9 desaturase activity	0.45 (0.35-1.43)	0.40 (0.04-0.59)	*0.0003*

Saturated Fatty Acids (Total SFA) = % C16:0 + % C18:0; Monounsaturated Fatty Acids (Total MUFA) = % C16:1 + % 9c,C18:1 + % 11c,C18:1; ω-3 Polyunsaturated Fatty Acids = %EPA + %DHA; ω-6 Polyunsaturated Fatty Acids = %LA + %DGLA + %ARA; Polyunsaturated Fatty Acids (PUFA) = %LA + %DGLA + %ARA + %EPA + %DHA; SFA/MUFA = (% C16:0 + % C18:0)/(% C16:1 + % 9c,C18:1 + % 11c,C18:1); ω-6/ω-3 ratio = (%LA + %DGLA + %ARA)/ (%EPA + %DHA); PUFA balance = [(%EPA + %DHA) / Total PUFA] × 100; Unsaturation index (UI) = (%MUFA × 1) + (%LA × 2) + (%DGLA × 3) + (%ARA × 4) + (%EPA × 5) + (%DHA × 6); Peroxidation index (PI) = (%MUFA × 0.025) + (%LA × 1) + (%DGLA × 2) + (%ARA × 4) + (%EPA × 6) + (%DHA × 8); Elongase-6 activity (EI) = C18:0 / C16:0; Delta-9 desaturase activity (D9DI) = 9c,C18:1 / C18:0; Delta-6 desaturase activity (D6DI) = C20:3 / C18:2; Delta-5 desaturase (D5DI) = C20:4 / C20:3.

Supplementary Table S3. Median values with minimum and maximum in brackets of fatty acids, homeostasis indexes and enzyme activity indexes of CE dogs, divided in groups affected by food-responsive enteropathy (FRE), antibiotic-responsive enteropathy (ARE), immunosuppressant-responsive enteropathy (IRE) and non-responsive enteropathy (NRE). Fatty acids are obtained as fatty acid methyl esters (FAME) after membrane isolation, lipid extraction and derivatization. The values are expressed as percentage of the found quantity in μg/mL referred to the 10 fatty acids of the cluster (% rel. quant.) as total quantity (100%) resulting from the gas chromatographic analysis, using calibration and quantitation protocols and standard reference compounds for each FAME, as described in Materials and Methods. The GC peak areas of the 10 fatty acids cohort corresponds to ca. 97% of the total peak areas of the chromatogram.

**Variable**	**FRE (n=28)**	**ARE (n=5)**	**IRE/NRE (n=15)**	**P value**
Palmitic Acid	10.79 (4.91-13.94)	10.44 (10.08-12.65)	11.44 (7.19-13.13)	0.492
Stearic Acid	23.29 (20.27-27.90)	23.48 (22.07-28.11)	22.66 (20.15-28.14)	0.847
**Saturated Fatty Acids**	34.55 (29.26-41.63)	33.96 (32.37-40.76)	34.99 (30.89-39.87)	0.704
Palmitoleic Acid	0.27 (0.05-1.12)	0.22 (0.16-0.66)	0.40 (0.07-2.23)	0.527
Oleic Acid	9.33 (8.09-14.68)	9.99 (7.49-11.76)	9.98 (7.51-12.35)	0.737
Vaccenic Acid	2.09 (0.29-3.24)	2.01 (1.76-2.88)	2.10 (0.07-3.35)	0.955
**Monounsaturated Fatty Acids**	12.13 (8.44-16.62)	12.18 (9.75-14.35)	12.80 (9.81-16.06)	0.556
Linoleic Acid	13.19 (9.75-17.71)	14.47 (11.44-16.27)	12.62 (7.39-18.98)	0.382
Dihomo-gamma-linolenic Acid	1.63 (0.14-2.74)	1.93 (1.36-2.06)	1.60 (0.97-3.35)	0.425
Arachidonic Acid	36.56 (32.22-43.75)	35.15 (30.65-38.03)	35.17 (29.70-38.56)	0.200
ω-6 Polyunsaturated Fatty Acids	51.26 (44.77-57.10)	51.05 (45.61-57.87)	52.34 (44.42-57.33)	0.545
EPA	0.85 (0.11-2.00)	0.78 (0.35-1.03)	0.89 (0.17-1.61)	0.662
DHA	1.30 (0.44-3.63)	1.21 (0.51-1.45)	1.08 (0.42-2.35)	0.496
ω-3 Polyunsaturated Fatty Acids	2.35 (0.71-4.71)	1.99 (0.87-2.48)	2.03 (1.11-3.26)	0.404
**Polyunsaturated Fatty Acids**	53.34 (46.11-59.94)	53.04 (45.61-57.87)	52.34 (44.42-57.33)	0.493
SFA/MUFA	2.80 (2.04-3.89)	2.99 (2.36-3.31)	2.66 (2.09-3.56)	0.638
ω-6/ω-3	21.66 (10.12-72.68)	25.60 (21.24-58.25)	23.51 (14.45-42.29)	0.411
PUFA Balance	4.41 (1.35-8.99)	3.76 (1.68-4.49)	4.08 (2.31-6.47)	0.176
Unsaturation Index	202.7 (175.6-221.8)	207.6 (173.9-210.9)	196.1 (174.6-217.1)	0.131
Peroxidation Index	179.7 (153.2-203.0)	172.1 (149.8-187.3)	170.4 (150.6-194.7)	0.525
Elongase-6 activity	0.88 (0.67-1.65)	0.87 (0.73-1.08)	0.87 (0.68-1.45)	0.889

Delta-6 desaturase activity	8.45 (3.88-90.82)	7.19 (5.92-11.91)	7.05 (3.65-16.97)	0.579
Delta-5 desaturase activity	2.81 (1.99-4.09)	2.42 (2.34-2.68)	2.87 (1.70-5.20)	0.151
Delta-9 desaturase activity	0.40 (0.04-0.59)	0.37 (0.34-0.51)	0.42 (0.32-0.55)	0.612

EPA, eicosapentaenoic acid; DHA, docosahexaenoic acid: SFA, saturated fatty acid; MUFA, monounsaturated fatty acids; PUFA, polyunsaturated fatty acids; FRE, food-responsive enteropathy; ARE, antibiotic-responsive enteropathy; IRE, immunosuppressant-responsive enteropathy; NRE non-responsive enteropathy. Saturated Fatty Acids (Total SFA) = % C16:0 + % C18:0. Monounsaturated Fatty Acids (Total MUFA) = % C16:1 + % 9c,C18:1 + % 11c,C18:1. ω-3 Polyunsaturated Fatty Acids = %EPA + %DHA. ω-6 Polyunsaturated Fatty Acids = %LA + %DGLA + %ARA. Polyunsaturated Fatty Acids (PUFA) = = %LA + %DGLA + %ARA + %EPA + %DHA. SFA/MUFA = (% C16:0 + % C18:0)/(% C16:1 + % 9c,C18:1 + % 11c,C18:1). ω-6/ω-3 ratio = (%LA + %DGLA + %ARA)/ (%EPA + %DHA). PUFA balance = [(%EPA + %DHA) / Total PUFA] × 100. Unsaturation index (UI) = (%MUFA × 1) + (%LA × 2) + (%DGLA × 3) + (%ARA × 4) + (%EPA × 5) + (%DHA × 6). Peroxidation index (PI) = (%MUFA × 0.025) + (%LA × 1) + (%DGLA × 2) + (%ARA × 4) + (%EPA × 6) + (%DHA × 8). Elongase-6 activity (EI) = C18:0 / C16:0. Delta-9 desaturase activity (D9DI) = 9c,C18:1 / C18:0. Delta-6 desaturase activity (D6DI) = C20:3 / C18:2. Delta-5 desaturase (D5DI) = C20:4 / C20:3.

Supplementary Table S4. Median values with minimum and maximum in brackets of fatty acids, homeostasis indexes and enzyme activity indexes of dogs affected by chronic enteropathies without and with loss of protein across the intestine. Fatty acids are obtained as fatty acid methyl esters (FAME) after membrane isolation, lipid extraction and derivatization. The values are expressed in μg/mL as percentage of the found quantity referred to the 10 fatty acids of the cluster (% rel. quant.) as total quantity (100%) resulting from the gas chromatographic analysis, using calibration and quantitation protocols and standard reference compounds for each FAME, as described in Materials and Methods. The GC peak areas of the 10 fatty acids cohort corresponds to ca. 97% of the total peak areas of the chromatogram.

Variable	CE (Non-PLE)(n = 39)	PLE (n = 9)	P value
Palmitic Acid	10.82 (4.91-13.94)	11.49 (7.19-13.13)	0.419
Stearic Acid	23.35 (20.27-28.11)	22.38 (20.15-20.14)	0.839
**Saturated Fatty Acids**	34.68 (29.26-41.63)	33.77 (33.28-39.87)	0.942
Palmitoleic Acid	0.26 (0.05-1.45)	0.4785 (0.07-2.22)	0.208
Oleic Acid	9.3 (7.49-14.68)	10.43 (9.77-12.35)	0.076
Vaccenic Acid	2.05 (0.29-3.24)	2.409 (0.07-3.35)	0.321
**Monounsaturated Fatty Acids**	12.12 (8.44-16.62)	13.31 (10.58-16.06)	0.076
Linoleic Acid	13.14 (9.75-18.54)	12.62 (7.39-18.98)	0.839
Dihomo-gamma-linolenic Acid	1.645 (0.14-3.35)	1.46 (0.97-2.91)	0.819
Arachidonic Acid	36.31 (29.70-43.75)	35.24 (32.26-38.44)	0.232
ω-6 Polyunsaturated Fatty Acids	51.05 (44.03-57.10)	50.84 (43.22-54.19)	0.554
EPA	0.86 (0.11-2.00)	0.8203 (0.17-1.39)	0.232
DHA	1.21 (0.42-3.63)	1.27 (0.74-2.35)	0.875
ω-3 Polyunsaturated Fatty Acids	2.20 (0.71-4.71)	2.09 (1.1-3.26)	0.222
**Polyunsaturated Fatty Acids**	53.04 (45.61- 59.94)	52.47 (44.42-55.66)	0.288
SFA/MUFA	2.857 (2.04-3.88)	2.538 (2.08-3.19)	0.065
ω-6/ω-3	21.79 (10.12-72.68)	24.77(14.45-42.29)	0.696
PUFA Balance	4.38 (1.35-8.99)	3.88 (2.3-6.47)	0.682
Unsaturation Index	199.9 (173.9-221.8)	200.5 (174.6-207.0)	0.264
Peroxidation Index	178.8 (149.8-203.0)	177.7 (150.6-188.2)	0.259
Elongase-6 activity	0.87 (0.67-1.65)	0.93 (0.69-1.45)	0.415
Delta-6 desaturase activity	8.23 (3.65-90.82)	7.19 (5.73-12.40)	0.751

Delta-5 desaturase activity	2.80 (1.84-4.09)	2.74 (1.70-5.20)	0.840
Delta-9 desaturase activity	0.40 (0.04-0.59)	0.43 (0.37-0.55)	0.08

EPA, eicosapentaenoic acid; DHA, docosahexaenoic acid: SFA, saturated fatty acid; MUFA, monounsaturated fatty acids; PUFA, polyunsaturated fatty acids; CE, chronic enteropathy; PLE, protein-losing enteropathy. Saturated Fatty Acids (Total SFA) = % C16:0 + % C18:0. Monounsaturated Fatty Acids (Total MUFA) = % C16:1 + % 9c,C18:1 + % 11c,C18:1. ω-3 Polyunsaturated Fatty Acids = %EPA + %DHA. ω-6 Polyunsaturated Fatty Acids = %LA + %DGLA + %ARA. Polyunsaturated Fatty Acids (PUFA) = = %LA + %DGLA + %ARA + %EPA + %DHA. SFA/MUFA = (% C16:0 + % C18:0)/(% C16:1 + % 9c,C18:1 + % 11c,C18:1). ω-6/ω-3 ratio = (%LA + %DGLA + %ARA)/ (%EPA + %DHA). PUFA balance = [(%EPA + %DHA) / Total PUFA] × 100. Unsaturation index (UI) = (%MUFA × 1) + (%LA × 2) + (%DGLA × 3) + (%ARA × 4) + (%EPA × 5) + (%DHA × 6). Peroxidation index (PI) = (%MUFA × 0.025) + (%LA × 1) + (%DGLA × 2) + (%ARA × 4) + (%EPA × 6) + (%DHA × 8). Elongase-6 activity (EI) = C18:0 / C16:0. Delta-9 desaturase activity (D9DI) = 9c,C18:1 / C18:0. Delta-6 desaturase activity (D6DI) = C20:3 / C18:2. Delta-5 desaturase (D5DI) = C20:4 / C20:3.

Supplementary Table S5. Median values with minimum and maximum in brackets of fatty acids, homeostasis indexes and enzyme activity indexes of that responded to therapeutic trials and dogs that poorly or not responded (NRE). Fatty acids are evaluated as fatty acid methyl esters (FAME) after membrane isolation, lipid extraction and derivatization. The values are expressed in μg/mL as percentage of the found quantity referred to the 10 fatty acids of the cluster (% rel. quant.) as total quantity (100%) from the gas chromatographic analysis, using calibration and quantitation protocols and standard reference compounds for each FAME, as described in Materials and Methods. The GC peak areas of the 10 fatty acids cohort corresponds to ca. 97% of the total peak areas of the chromatogram. Italic values denote statistical significance.

Variable	RE (n = 45)	NRE (n = 3)	P value
Palmitic Acid	10.82 (4.91-13.94)	11.73 (11.25-12.81)	0.282
Stearic Acid	23.35 (20.15-28.11)	22.34 (22.3-28.14)	0.936
**Saturated Fatty Acids**	34.43 (29.26-41.63)	35.15 (33.55-39.87)	0.462
Palmitoleic Acid	0.33 (0.05-2.22)	0.25 (0.21-1.06)	0.747
Oleic Acid	9.37 (7.49-14.68)	12.14 (9.42-12.35)	0.074
Vaccenic Acid	2.06 (0.07-3.24)	2.69 (2.64-3.35)	*0.007*
**Monounsaturated Fatty Acids**	12.14 (8.44-16.62)	15.71 (12.38-16.06)	*0.031*
Linoleic Acid	13.14 (9.75-18.98)	9.97 (7.39-14.19)	0.170
Dihomo-gamma-linolenic Acid	1.6 (0.14-3.35)	1.29 (0.97-1.40)	0.060
Arachidonic Acid	35.90 (29.70-43.75)	35.24 (32.26-38.44)	0.688
ω-6 Polyunsaturated Fatty Acids	51.05 (44.03-57.10)	47.13 (43.22-50.84)	0.054
EPA	0.85 (0.11-2.00)	0.63 (0.20-1.05)	0.303
DHA	1.27 (0.42-3.63)	1.00 (0.99-2.21)	0.968
ω-3 Polyunsaturated Fatty Acids	2.20 (0.71-4.71)	1.63 (1.19-3.26)	0.629
**Polyunsaturated Fatty Acids**	53.08 (45.61-59.94)	50.4 (44.42-52.47)	0.060
SFA/MUFA	2.80 (2.04-3.88)	2.53 (2.08-2.84)	0.841
ω-6/ω-3	21.79 (10.12-72.68)	31.06 (14.45-36.11)	0.132
PUFA Balance	4.38 (1.35-9.99)	3.11 (2.69-6.47)	0.811
Unsaturation Index	200.5 (173.9-221.8)	195.1 (174.6-207.0)	0.284
Peroxidation Index	178.8 (149.8-203.0)	170.1 (150.6-188.2)	0.411
Elongase-6 activity	0.87 (0.67-1.65)	1.04 (0.74-1.10)	0.511
Delta-6 desaturase activity	8.20 (3.65-90.82)	10.11 (5.73-10.21)	0.714
Delta-5 desaturase activity	2.77 (1.70-4.09)	3.23 (2.48-5.20)	0.216
Delta-9 desaturase activity	0.40 (0.04-0.59)	0.43 (0.42-0.55)	0.146

EPA, eicosapentaenoic acid; DHA, docosahexaenoic acid: SFA, saturated fatty acid; MUFA, monounsaturated fatty acids; PUFA, polyunsaturated fatty acids; RE, responsive enteropathy; NRE non-responsive enteropathy. Saturated Fatty Acids (Total SFA) = % C16:0 + % C18:0. Monounsaturated Fatty Acids (Total MUFA) = % C16:1 + % 9c,C18:1 + % 11c,C18:1. ω-3 Polyunsaturated Fatty Acids = %EPA + %DHA. ω-6 Polyunsaturated Fatty Acids = %LA + %DGLA + %ARA. Polyunsaturated Fatty Acids (PUFA) = = %LA + %DGLA + %ARA + %EPA + %DHA. SFA/MUFA = (% C16:0 + % C18:0)/(% C16:1 + % 9c,C18:1 + % 11c,C18:1). ω-6/ω-3 ratio = (%LA + %DGLA + %ARA)/ (%EPA + %DHA). PUFA balance = [(%EPA + %DHA) / Total PUFA] × 100. Unsaturation index (UI) = (%MUFA × 1) + (%LA × 2) + (%DGLA × 3) + (%ARA × 4) + (%EPA × 5) + (%DHA × 6). Peroxidation index (PI) = (%MUFA × 0.025) + (%LA × 1) + (%DGLA × 2) + (%ARA × 4) + (%EPA × 6) + (%DHA × 8). Elongase-6 activity (EI) = C18:0 / C16:0. Delta-9 desaturase activity (D9DI) = 9c,C18:1 / C18:0. Delta-6 desaturase activity (D6DI) = C20:3 / C18:2. Delta-5 desaturase (D5DI) = C20:4 / C20:3.

2. Material

For the membrane work-up and fatty acid methyl ester (FAME) transformation the materials with the corresponding suppliers are indicated here below:

Materials	Company
n-Hexane 95%	TITOLCHIMICA, Pontecchio Polesine (Ro) Italy
Methyl alcohol HPLC	TITOLCHIMICA Pontecchio Polesine (Ro) Italy
Chloroform extra pure 99.5%	TITOLCHIMICA Pontecchio Polesine (Ro) Italy
PBS pH 7,4 RS	Carlo Erba, Milan (Italy)
Polar Lipid Mixture (quantitative)	MATREYA LLC State College, PA, USA
non-Polar Lipid Mixture B (quantitative)	MATREYA LLC State College, PA,USA
Phosphatidylserine	MATREYA LLC State College, PA, USA
L-α-Phosphatidylcholine	Merck, Darmstadt, Germany
ALUGRAM Xtra sheets 200×200mm	Carlo Erba, Milan Italy
Potassium hydroxide, pellets RPE - For analysis	Carlo Erba, Milan Italy
Sodium sulfate anhydrous RS - For anhydrification	Carlo Erba, Milan Italy
C16:0 – palmitic acid methyl ester	Merck, Darmstadt Germany
C16:1 – palmitoleic acid methyl ester	Merck, Darmstadt Germany
C18:0 – stearic acid methyl ester	Supelco, Bellefonte, PA, USA
9c, C18:1 – oleic acid methyl ester	Merck, Darmstadt, Germany
11c, C18:1 – vaccenic acid methyl ester	Supelco, Bellefonte, PA ,USA
LA omega-6 – C18:2 – linoleic acid methyl ester	Merck, Darmstadt Germany
DGLA omega-6 – C20:3 dihomogammalinolenic acid methyl ester	Merck, Darmstadt Germany
ARA omega-6 C20:4 – arachidonic acid methyl ester	Merck, Darmstadt Germany
EPA omega-3 – C20:5 – eicosapentaenoic acid methyl ester	Supelco, Bellefonte, PA, USA
DHA omega-3 – C22:6 – docosahexaenoic acid methyl ester	Merck, Darmstadt Germany
Supelco 27 component FAME mix	Supelco, Bellefonte, PA, USA

Materials were used as received.

The analytical procedure followed the steps represented in **Figure S1**.

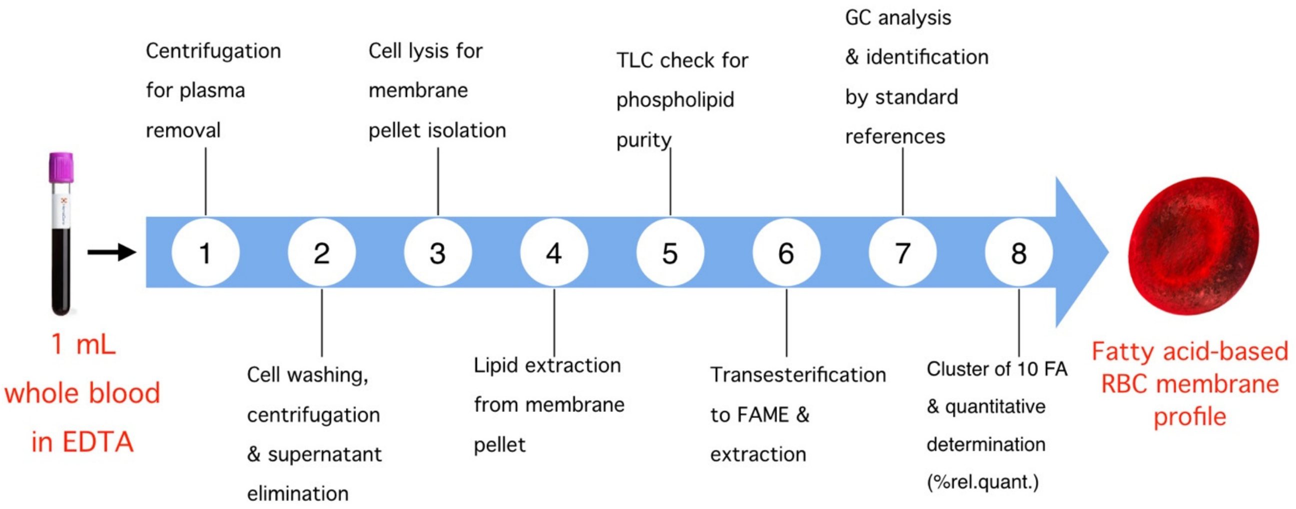

Supplementary Figure S1. Steps of the laboratory procedure from the blood sample in EDTA to the fatty acid-based RBC membrane profile.

3. Details of the GC analysis of FAME – Calibration procedure

In this paper we proceeded with the determination of the previously reported benchmark for membrane fatty acid profile [29], analyzing a cluster of 10 fatty acids, which also corresponds to chromatographic peak areas >97% (see representative GC run reported in Supplementary Figure 2). This cluster consists of: 2 saturated fatty acids (SFA: palmitic and stearic acids); 3 monounsaturated fatty acids (MUFA, palmitoleic, oleic and cis-vaccenic acids); 3 polyunsaturated fatty acids omega-6 (PUFA, linoleic, dihomo-gamma linolenic, arachidonic acids); 2 polyunsaturated fatty acids omega-3 (PUFA, eicosapentaenoic and docosahexaenoic acids) as shown in Table 3 of the main text.

The quantitation of the fatty acids was carried out by calibration procedures, for which the following protocol has been followed:

initially a n-hexane (HPLC grade, Titolchimica) 5mM solution of stearic acid methyl ester (2 mg in 1340 µL) was prepared and 1µl was directly injected to the Agilent 7890B GC system equipped with a flame ionization detector and a DB-23 (50%-Cyanopropyl)-methylpolysiloxane capillary column (60 m, 0.25 mm i.d., 0.25 µm film thickness, Agilent, Milan). The following oven conditions were established to be kept for all the analyses: the initial temperature was 165 °C, held for 3 min, followed by an increase of 1 °C/min up to 195 °C, held for 40 min, followed by a second increase of 10 °C/min up to 240 °C, held for 10 min. The carrier gas was hydrogen, held at a constant pressure of 16.482 psi. The injections were repeated in triplicates.

The second round of injections for calibration was then performed with 0.5 mM solution of the same fatty acid methyl ester (taking 100µL of the initial solution and diluting with 900µL of n-hexane), injecting 1 µL as previously described for triplicates.

The same protocol was carried out using dilutions of 0.05mM, 0.005mM and 0.0005mM of stearic acid methyl ester.

In all the injections a calibration curve was created using the software of the GC equipment (Agilent 7890B GC system, Agilent, Milan). Using the concentration of 0.0005mM for methyl stearate, the corresponding peak area was detectable but not quantifiable, indicating this concentration as the limit of detection (LOD) of the specific GC system (<0.5nM).

The above protocol has been followed for all the fatty acids of the cohort.

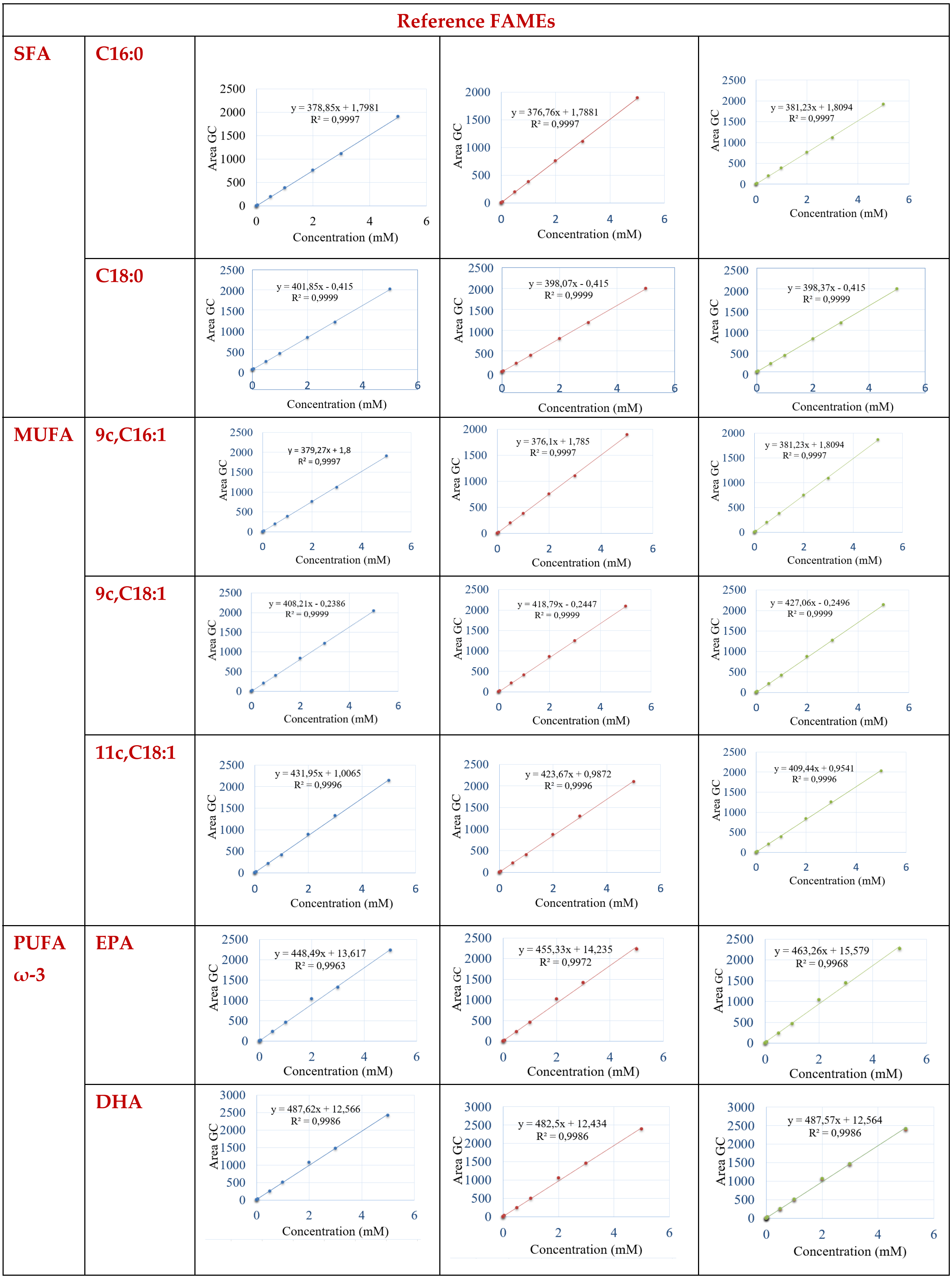
Reference FAMEs
SFA
C16:0
y = 378,85x + 1,7981
R² = 0,9997
y = 376,76x + 1,7881
R² = 0,9997
y = 381,23x + 1,8094
R² = 0,9997
C18:0
y = 401,85x - 0,415
R² = 0,9999
y = 398,07x - 0,415
R² = 0,9999
y = 398,37x - 0,415
R² = 0,9999
MUFA
9c,C16:1
y = 379,27x + 1,8
R² = 0,9997
y = 376,1x + 1,785
R² = 0,9997
y = 381,23x + 1,8094
R² = 0,9997
9c,C18:1
y = 408,21x - 0,2386
R² = 0,9999
y = 418,79x - 0,2447
R² = 0,9999
y = 427,06x - 0,2496
R² = 0,9999
11c,C18:1
y = 431,95x + 1,0065
R² = 0,9996
y = 423,67x + 0,9872
R² = 0,9996
y = 409,44x + 0,9541
R² = 0,9996
PUFA
ω-3
EPA
y = 448,49x + 13,617
R² = 0,9963
y = 455,33x + 14,235
R² = 0,9972
y = 463,26x + 15,579
R² = 0,9968
DHA
y = 487,62x + 12,566
R² = 0,9986
y = 482,5x + 12,434
R² = 0,9986
y = 487,57x + 12,564
R² = 0,9986
Area GC
Concentration (mM)

PUFA ω-6

LA

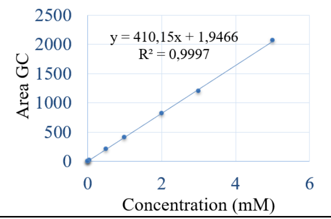

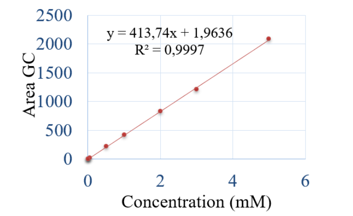

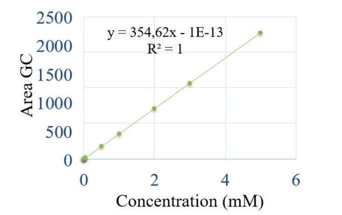

DGLA

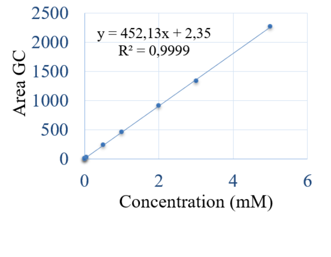

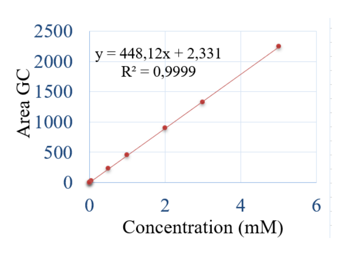

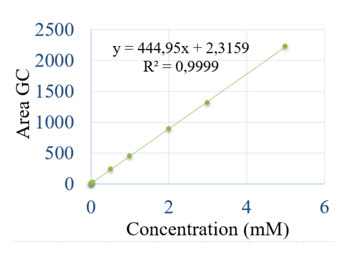

ARA

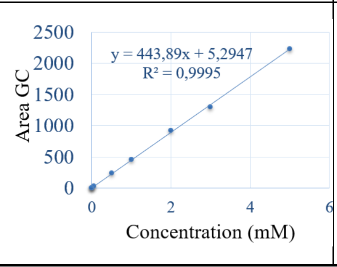

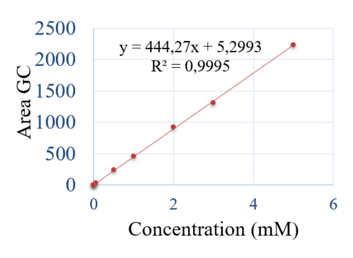

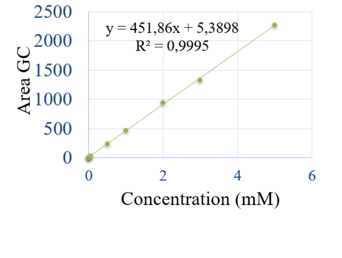

Reference FAMEs

SFA

C16:0

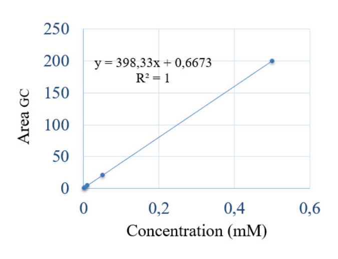

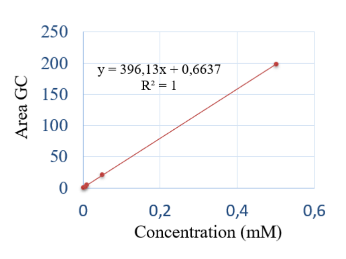

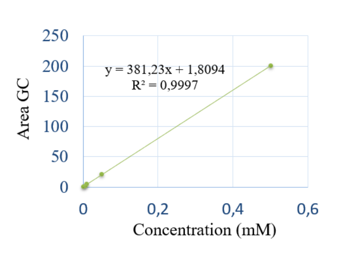

C18:0

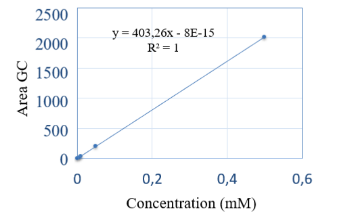

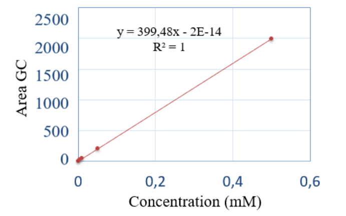

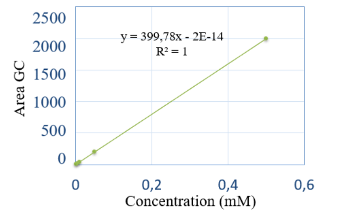

MUFA

9c,C16:1

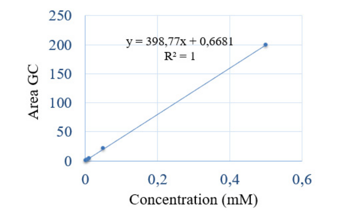

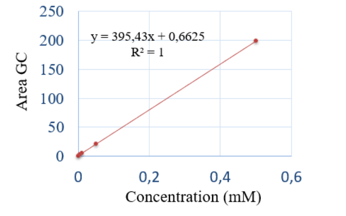

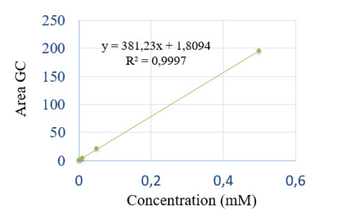

9c,C18:1

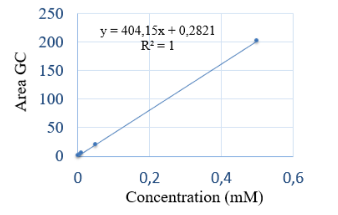

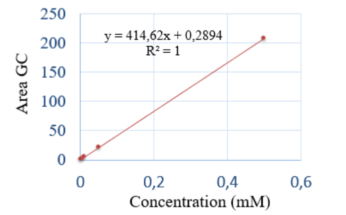

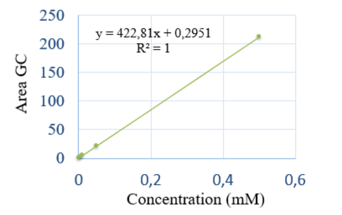

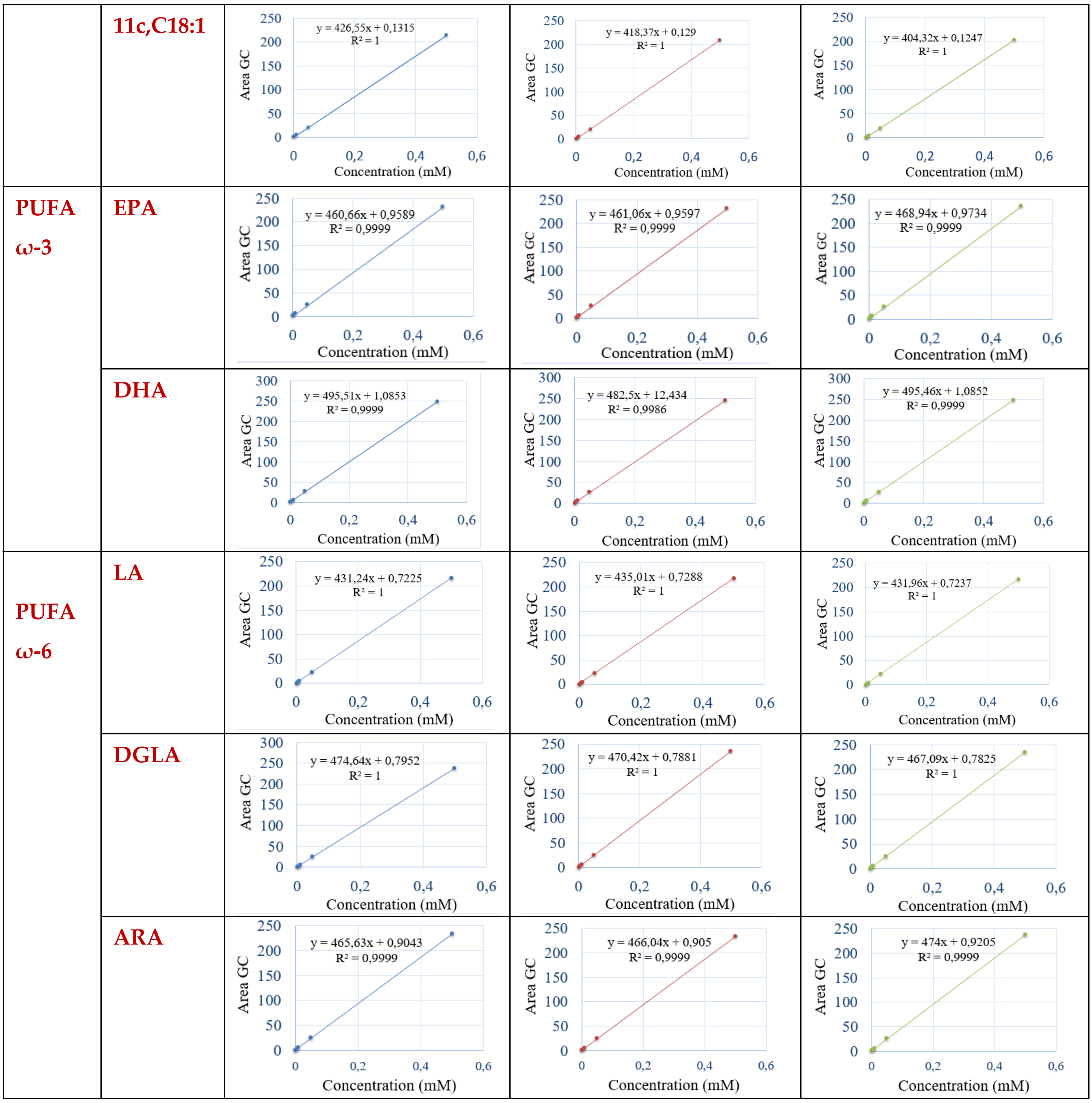

Supplementary Figure S2. Calibration curves of the 10 fatty acids at high (0.5-5 mM) and low (from 0.001 mM to 0.5 mM) concentration ranges, chosen as representatives of the SFA, MUFA and PUFA families present in the erythrocyte membrane phospholipids.

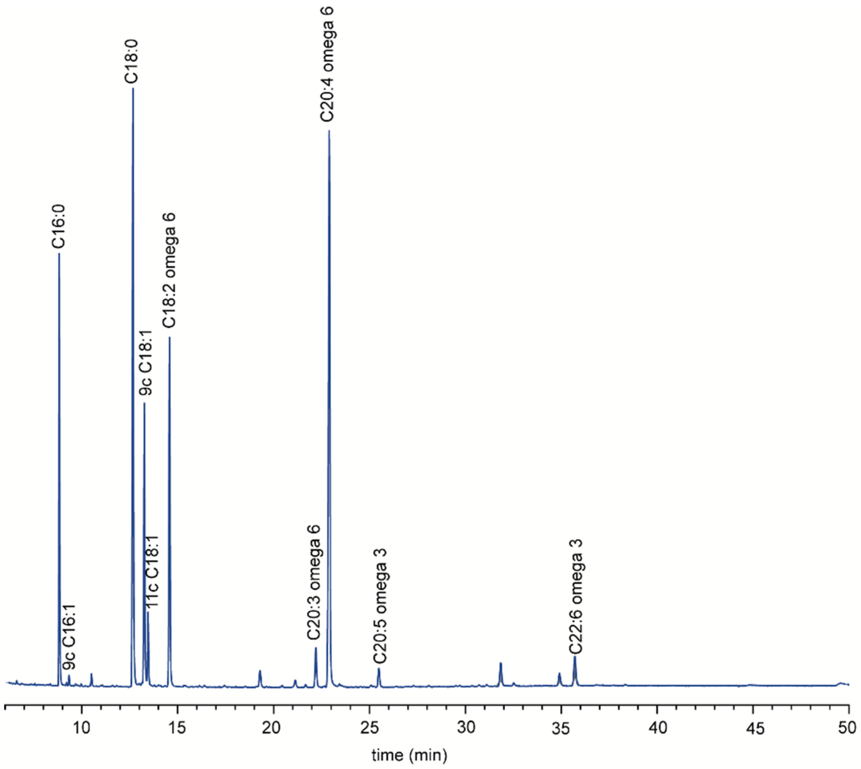

Supplementary Figure S3. Representative GC chromatogram of the FAME obtained from dog erythrocyte membrane phospholipids after work-up, as described in the main text. The 10 fatty acids chosen for the cluster are satisfactorily separated and recognized by appropriate standard references. The sum of their areas corresponds to >97% of the total peak areas.

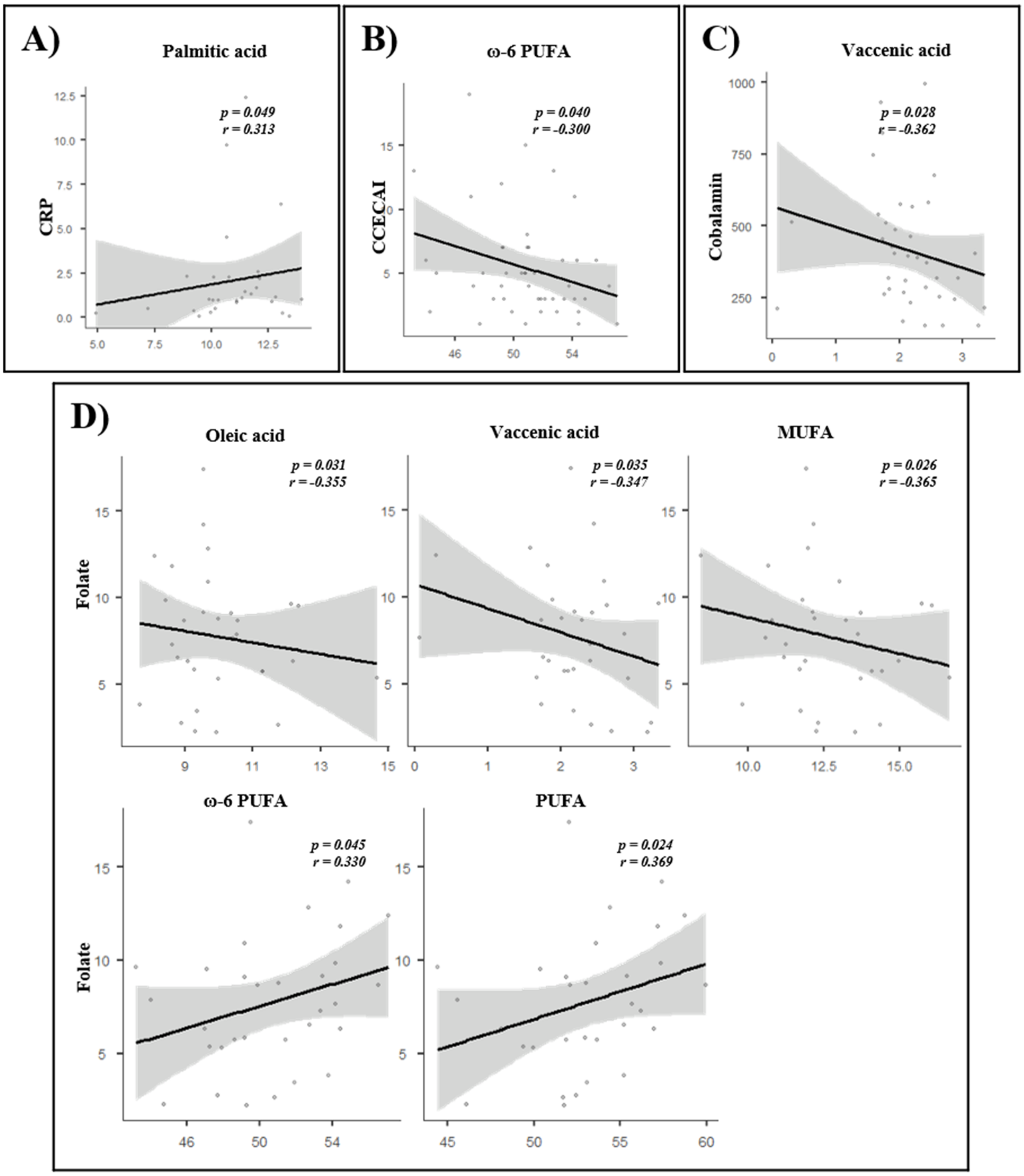

Supplementary Figure S4. Spearman rank correlation between: (A) C-reactive protein and palmitic acid, (B) the Canine Chronic Enteropathy Clinical Activity Index and ω-6 polyunsaturated fatty acids, (C) cobalamin and vaccenic acid, (D) folate and oleic acid, vaccenic acid, total monounsaturated fatty acids, ω-6 polyunsaturated fatty acids and total polyunsaturated fatty acids. CRP, C-reactive protein; CCECAI, Canine Chronic Enteropathy Clinical Activity Index; MUFA monounsaturated fatty acids; PUFA, polyunsaturated fatty acids.

Reference

29. Prasinou, P.; Crisi, P.E.; Chatgilialoglu, C.; Di Tommaso, M.; Sansone, A.; Gramenzi, A.; Belà, B.; De Santis, F.; Boari, A.; Ferreri, C. The Erythrocyte Membrane Lipidome of Healthy Dogs: Creating a Benchmark of Fatty Acid Distribution and Interval Values. *Front Vet Sci* **2020**, *7*, 502, doi:10.3389/fvets.2020.00502.
